# CD56-negative NK cells: Frequency in peripheral blood, expansion during HIV-1 infection, functional capacity, and KIR expression

**DOI:** 10.3389/fimmu.2022.992723

**Published:** 2022-09-23

**Authors:** Alexander T. H. Cocker, Fuguo Liu, Zakia Djaoud, Lisbeth A. Guethlein, Peter Parham

**Affiliations:** ^1^ Department of Structural Biology and Department of Microbiology and Immunology, Stanford University School of Medicine, Stanford, CA, United States; ^2^ Laboratory Koch Institute for Integrative Cancer Research, Massachusetts Institute of Technology, Cambridge, MA, United States; ^3^ Department of Biochemistry, Microbiology and Immunology, University of Ottawa, Ottawa, ON, Canada; ^4^ Children’s Hospital of Eastern Ontario Research Institute, Ottawa, ON, Canada

**Keywords:** NK cells, innate immunity, CD56neg, chronic infection, meta-analysis

## Abstract

Human NK cells are usually defined as CD3^-^CD56^+^ lymphocytes. However, a CD56^-^CD16^+^ (CD56neg) lymphocyte population that displays NK-associated markers expands during chronic viral infections such as HIV-1 and HCV, and, to lesser extent, in herpesvirus infections. This CD56neg NK cell subset has been understudied because it requires the exclusion of other lymphocytes to accurately identify its presence. Many questions remain regarding the origin, development, phenotype, and function of the CD56neg NK cell population. Our objective was to determine the frequency of this NK subset in healthy controls and its alteration in viral infections by performing a meta-analysis. In addition to this, we analyzed deposited CyTOF and scRNAseq datasets to define the phenotype and subsets of the CD56neg NK cell population, as well as their functional variation. We found in 757 individuals, from a combined 28 studies and 6 datasets, that the CD56neg subset constitutes 5.67% of NK cells in healthy peripheral blood, while HIV-1 infection increases this population by a mean difference of 10.69%. Meta-analysis of surface marker expression between NK subsets showed no evidence of increased exhaustion or decreased proliferation within the CD56neg subset. CD56neg NK cells have a distinctive pattern of KIR expression, implying they have a unique potential for KIR-mediated education. A perforin^-^CD94^-^NKG2C^-^NKp30^-^ CD56neg population exhibited different gene expression and degranulation responses against K562 cells compared to other CD56neg cells. This analysis distinguishes two functionally distinct subsets of CD56neg NK cells. They are phenotypically diverse and have differing capacity for education by HLA class-I interactions with KIRs.

## Introduction

Twelve years after the characterization of CD56 (alternatively named NCAM-1, N901, NKH-1 and Leu-19) as a defining marker of human natural killer (NK) cells (1), Hu et al. found that human immunodeficiency virus-1 positive (HIV-1^+^) individuals were deficient for CD56^+^CD16^+^ (CD56dim) NK cells (2). These individuals also exhibited an expanded subset of live lymphocyte-sized cells having the CD3^-^CD5^-^CD14^-^CD19^-^CD33^-^CD56^-^CD16^+^ surface phenotype. With this profile, these cells were clearly not T cells, B cells, monocytes, nor dendritic cells (DC). In addition, a decrease in CD56dim NK cell absolute count combined with a larger CD56^-^CD16^+^ (CD56neg) population proportion, supported Hu et al’s conclusion that CD56neg cells comprise a distinctive subset of NK cells (2). Furthermore, the CD56neg subset expresses killer cell immunoglobulin-like receptors (KIRs), among other NK related markers (3–6). Functionally, the CD56neg population exhibited a reduced lytic response compared to CD56dim NK cells from the same donor. This feature is consistent with reports that CD56neg cells have poor major-histocompatibility complex (MHC)-unrestricted cytotoxicity (7, 8). NK cell immunity is regulated by signaling *via* receptors for human leukocyte antigen (HLA) class-I, including KIRs, therefore altered expression of HLA class-I or KIRs can disrupt NK cell immune functions (9). Lacking the classical NK cell functional and phenotypic identifiers of the CD56dim and CD56^high^CD16^-^ (CD56bright) NK cell subpopulations, CD56neg NK cells have yet to be studied in detail.

McKenzie and colleagues reported a population of CD56^-^CD16^+^ large granular lymphocytes that lacked expression of CD3, CD4, CD8, CD14, CD19, CD25 and HLA-DR in cancer patients (10). Earlier studies by Yu, Ellis, and Lanier also described a CD56^-^CD16^+^ NK cell subset (11–13), with the latter estimating that it comprised <5% of CD16^+^ lymphocytes, although other cell populations were not excluded in these studies. A phenotypically similar population of cells had been noted in an exploration of NK cell development in human fetal tissue isolates (14). Cord blood and fetal liver cells with the CD56^-^CD16^+^ phenotype were observed following the depletion of CD3, CD4, CD5, CD14, CD19, CD33 and CD71 expressing cells, with these authors noting that further investigation of this population and how it relates to NK cells was warranted (14). More recently, Bozzano et al. defined CD56neg NK cell heterogeneity based on the expression of perforin, CD94, NKG2C, NKp30, CD57 and DNAM-1, whereas Hong et al. used CD122, CCR7 and CD57 expression (15, 16). CD7 expression has been used as a marker of NK cells that splits the CD56neg population, and NKp80 has more recently been seen to play a similar role (17–19).

NK ontogenesis can be defined as a linear development from precursor cells (CD122^+^CD34^+^CD38^+^CD123^-^CD45RA^+^CD7^+^CD10^+^CD127^-^) to CD56bright cells (CD56^high^NKG2A/C^+^NKp30^+^NKp46^+^), then CD56dim cells (CD56^+^CD16^+^), and ending their maturation by gaining KIRs and CD57 expression (20). NK diversity however is more complex than this, with 6,000 to 30,000 distinct NK phenotypes being estimated to exist in one individual (21). Infections are also associated with deviations from linear NK ontogenesis. Cytomegalovirus (CMV) is linked to an expansion of a CD56^+^CD16^+^NKG2C^+^CD57^+^FcεR1γ^-^ NK subset, *Mycobacterium tuberculosis* and malaria associated with expanded CD45RO^+^ NK populations, and Influenza A with CD49a^+^CD16^-^CXCR3^+^ NK cells (22). Furthermore, Bozzano et al. identified an unconventional CD34^-^CD56^-^CD16^+^perforin^-^CD94^-^CXCR4^+^ precursor that can generate CD56^+^CD16^+^NKG2C^+^ NK cells (16).

In non-human primates the expression of CD8, NKG2A, NKG2C, NKp30 and NKp46 are used to define NK cells and their developmental stages instead of CD56, which is expressed to a lesser degree than in human NK cells (23–25). Human NK cell expression of CD56 correlates with NK cell motility, formation of a developmental synapse, and control of cytotoxicity (26, 27). Unlike humans, chimpanzee and macaque CD56^-^ cells account for a large proportion of peripheral blood NK cells (28, 29). Despite this difference, macaque NK cells that lack CD56 have been considered equivalent to human CD56dim NK cells in their maturation and function (30, 31).

The trend towards greater CD56 expression by human peripheral blood NK cells suggests this NK phenotype is more recently derived, indicating the CD56neg subset is more closely related to NK cells found in ancestral primates. The extensive divergence of the cognate MHC class-I ligands and KIRs among humans and non-human primates demonstrates that strong selective forces have immunologically diversified these species (32). The concept that the genetic diversity of KIRs and MHC class-I has been driven by a long history of viral infections, as well as the increasing number of studies reporting associations between CD56neg NK cells and viruses (2, 33–36), suggest that this enigmatic NK population may contribute to the management and amelioration of chronic infections (37–39).

In their 2010 review, Björkström et al. framed a number of open questions regarding the CD56neg subset of NK cells: 1) Are CD56neg NK cells found in tissues outside of peripheral blood? 2) What is the maturation state of the CD56neg subset? 3) As CD56neg cells express KIRs, and are thus likely educated, what mechanism underlies their reduced functionality? 4) Are CD56neg cells exhausted or does their phenotype reflect a unique function? And 5), are the expanded CD56neg cell populations present in chronic viral infections different from the CD56neg cells in uninfected individuals (40)? In this paper we aimed to address these questions by performing a meta-analysis of the published literature on CD56neg NK cell studies. Additionally, we hypothesized that, following the recent publication describing two populations of CD56neg NK cells by Bozzano et al. (16), we could identify phenotypically and functionally distinct subsets of CD56neg NK cells through analysis of deposited CyTOF and single-cell RNA sequencing (scRNAseq) datasets.

## Methods

### Meta-analysis

Methods for meta-analysis were based on the Cochrane guidelines, and presented following the MOOSE criteria for reporting meta-analysis of observational studies (**Supplementary Table 1**). Data were extracted from the published studies and imported into the R package, meta (41). Random effect model (REM) assessment, using the Sidik-Jonkman estimator and the Hartung-Knapp-Sidik-Jonkman method, were used for meta-analysis because they assume that both the study methodology and the cohorts studied are highly variable. REM analysis was undertaken on all extracted data. Analysis was also done after excluding data extracted using median and IQR, to determine if their inclusion affected the results (**Supplementary Table 2**).

### Deposited dataset analysis

The Flowrepository and Immport dataset repository websites (flowrepository.org and immport.org) were searched for CyTOF datasets that included NK cell markers defining CD56neg cells, as well as those that detect and distinguish KIRs. Datasets were chosen to include markers that met our minimum criteria for CD56neg NK cell definition, live cells with CD3^-^CD14^-^CD19^-^CD56^-^CD16^+^ phenotype. Samples were gated to identify the CD56neg NK cells. Only samples containing 100 or more CD56neg NK cells were analyzed further. Backgating and different cell lineages within the datasets were used to determine appropriate gates for the extraction of single marker expression in Flowjo v10.4.2. The datasets analyzed and the details of population gating are included in the supplementary materials (**Supplementary Figures 1–7**).

### Single cell RNAseq analysis of the NK cells obtained from an HIV-1^+^ donor

Bradley et al. (33) performed scRNAseq on NK cells obtained from an HIV-1^+^ donor. We accessed and analyzed their data using the R package Seurat v3.6.3 (33, 42). Excluded from analysis were cell doublets, dead cells, and empty droplet outliers displaying an unusually high (>4000) RNA content, a high content of mitochondrial genes (>10%), or a low number (<200) of total genes detected. The data were then normalized by dividing the number of genes by the total RNA and log transformed. Variable features were identified, gene expression scaled, and dimension reduction applied. The dimensionality of the data was determined through resampling/bootstrapping, with the result being used to denote the expected number of clusters for the neighbor analysis. Clusters were assessed for expression of the markers (perforin, CD94, NKG2C, CD57, NKp30, and DNAM-1) that Bozzano et al. (16) associated with a specific CD56neg NK cell subset. Meta-clusters were generated by combining CD56neg NK cell clusters with the corresponding gene expression of these markers. The commands used to complete this R analysis are included in the supplementary materials (Supplementary File 1). The webtool g:Profiler (43) (https://biit.cs.ut.ee/gprofiler/gost) was used to determine possible identities of clusters generated by differential gene expression analysis. The top 50 differentially expressed genes from clusters were assessed by running an ordered g:GOSt query using Gene Ontology biological process (BP), cell components (CC), and KEGG biological pathway data sources. Bonferroni correction was applied and a threshold of 0.001 set.

### Statistical analysis

We treated CyTOF data as being non-parametric, making no assumptions about the data distribution. Comparison of between group data was done in Prism v9 with ANOVA using Kruskal-Wallis tests corrected for false discovery rate (FDR) with the Benjamini, Krieger and Yekutieli (BKY) method, or Mann-Whitney where only two groups were compared. Within group analysis was undertaken with ANOVA using Freidman tests corrected for FDR with the BKY method. Spearman’s correlation corrected for FDR using the Benjamini and Hochberg method was used to generate the HLA correlation network in R with the qgraph package (44) (**Supplementary File 2**).

## Results

### Meta-analysis of publications including CD56neg NK cells

**Figure 1:**
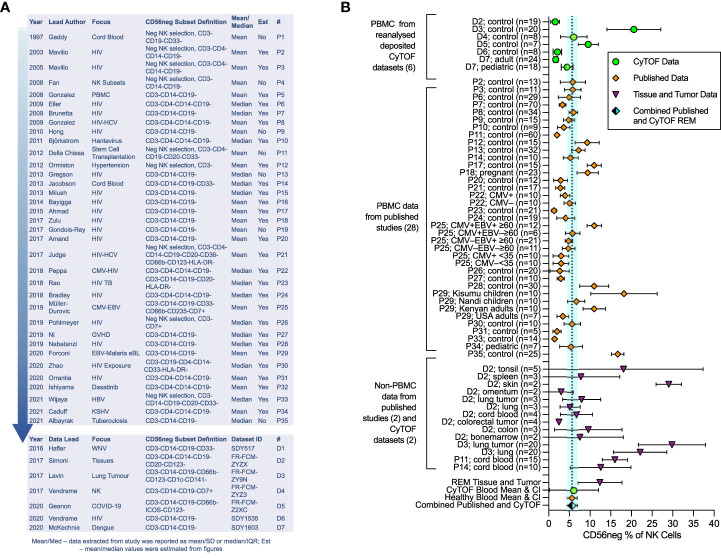
Meta-analysis of CD56neg subset frequency in NK cells from published studies and deposited datasets. Results from the meta-analysis of published and extracted data from CyTOF datasets on CD56neg NK cells. **(A)** 35 papers met the minimum CD56neg NK cell definition and had extractable data. Year of publication, first author’s last-name, the publication focus, and CD56neg definition are listed. The last columns show if mean/SD or median/IQR were extracted, and if data values were estimated from figures. The # column provides a key for identifying the papers in the graphs. Analyzed CyTOF datasets are also shown. **(B)** CD56neg subset % of NK cells in the peripheral blood from published studies (REM mean 5.65%, CI ± 1.21, I2 = 97%; study n = 28, combined sample n = 653), from CyTOF datasets (REM mean 6.11%, CI ± 5.92, I2 = 93%; dataset n = 6, combined sample n = 104), and combined published and CyTOF datasets, the mean and range of which are shown as a dashed line and blue shaded column respectively (REM mean 5.67%, CI ± 1.22, I2 = 96%, combined sample n = 757). CD56neg subset frequency in tissue resident NK cells is shown, including both healthy tissue and tumors (REM mean 12.38%, CI ± 5.32, I2 = 97%; study n = 2, dataset n = 2, combined sample n = 96).

Pubmed was searched for any paper that mentioned “CD56neg NK cells” or “CD56^-^CD16^+^ NK cells”, returning 31 and 367 results respectively. We identified and assessed papers that specifically explored the CD56neg subset, while works not pertaining to CD56neg cells in humans, or reviews, were excluded. One hundred and thirteen primary research papers were found. As study definitions of CD56neg cells varied, we excluded studies from our analysis that did not remove CD3, CD14 and CD19 expressing cell populations through cytometric gating or NK cell enrichment. Of the 113 studies, 50 met this minimum definition of CD56neg cells (CD3^-^CD14^-^CD19^-^CD56^-^CD16^+^), and after excluding results from cultured cells or transplant recipients as possible confounders for meta-analysis, data could be extracted from 35 papers (**Figure 1A**) (3–6, 15, 17–19, 33, 35, 36, 45–68). Where mean and standard deviation (SD) were not explicitly stated in the manuscript, they were estimated from figures using the WebPlotDigitizer tool (69). Where median and interquartile range (IQR) were used, we treated the data as normally distributed, with the median used as the mean and the IQR being divided by 1.35 to estimate the SD following published methods (70). CyTOF human datasets with markers to define CD56neg NK cells were identified in the Immport.org and Flowrepository.org public repositories.

### The CD56neg subset is a stable population of peripheral blood NK cells

We undertook meta-analysis of published studies and of analyzed deposited CyTOF datasets, with the aim of defining the average frequency of CD56neg cells in healthy blood and in tissues, determining the extent of HIV-1-mediated expansion of the subset, and confirming the variation in functional response to HLA-negative target cells. Our analysis exploring published studies shows that the CD56neg subset represents 5.65% of NK cells in the peripheral blood (CI ± 1.21, I^2^ = 97%) (**Figure 1B**), 7.40% (CI ± 5.00, I^2^ = 93%) when assessing analyzed CyTOF datasets (71–77), and 5.67% (CI ± 1.22, I^2^ = 96%) when combining published and CyTOF data (**Figure 1B**). High study heterogeneity was observed, which we expected from these observational studies on a variety of cohorts. Our results are consistent with the individual findings of both the Müller-Durovic and Forconi studies (35, 47). The former found Cytomegalovirus and Epstein-Barr virus dual infected (CMV^+^EBV^+^) >60 year old individuals had increased frequencies of CD56neg NK cells compared to age matched or younger groups with single EBV or CMV infection status (35). This group was similarly found to have a higher CD56neg subset frequency than the combined REM mean. Likewise, Kenyan donors in the Forconi et al. study had a higher frequency of CD56neg NK cells than the combined REM mean, whereas participants from the USA did not (47), suggesting that differences in environment and genetics could be influential in this variation.

Data for tissue resident CD56neg NK cells is limited. We found two papers and two publicly available datasets with extractable non-peripheral blood CD56neg NK cell frequencies (5, 53, 75, 76). The CD56neg cell subset comprises 12.38% (CI ± 5.32, I^2^ = 97%) of NK cells in tissues and tumors (**Figure 1B**). One question raised by the Björkström review (40) asked if CD56neg NK cells are present outside of peripheral blood. Our analysis here suggests that this is the case. CD56neg cell frequency in tonsils was very variable, a limitation due to the small sample number and possibly of the tissue disaggregation process. However, proportions of CD56neg NK cells in skin and lung were higher than in blood, indicating that CD56neg NK cells may be found in increased proportion at sites of greater exposure to externally derived antigens. This is also the case in cord blood where maternal immune responses are finely balanced to tolerate fetal antigens. Whether CD56neg NK cells are involved in tolerance, promoting or inhibiting responses to external antigens, or have a role in tissue maintenance is not known.

### NK cell marker frequency is lower in the CD56neg subset compared to CD56dim cells

**Figure 2:**
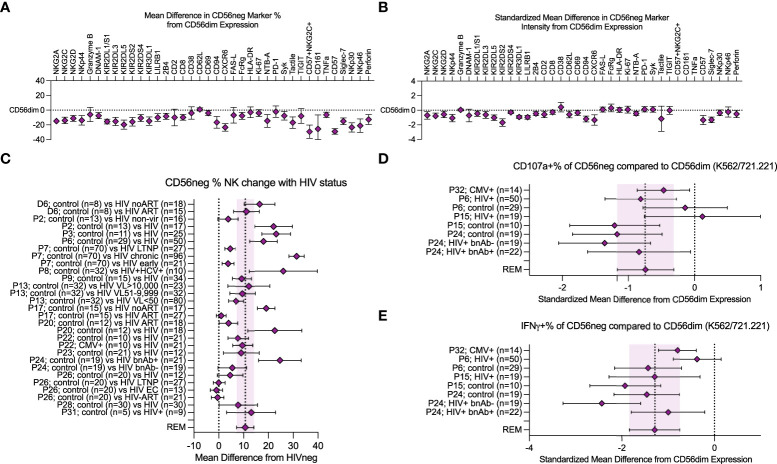
Meta-analysis of CD56neg subset frequency in HIV-1^+^ individuals, and variation in phenotype and function. **(A)** combined mean difference in CD56neg subset frequency of markers compared to paired CD56dim cells are shown at zero. **(B)** combined standardized mean difference in expression intensity for marker expressing CD56neg cells in comparison to the CD56dim subset. **(C)** mean difference of CD56neg % NK cells in peripheral blood in HIV-1^+^ groups from HIV-1^-^ controls (REM mean difference 10.69%, CI ± 3.34, I2 = 95%; study n = 15, dataset n = 1, combined HIV-1^+^ samples n = 751, combined HIV-1^-^ samples = 354). **(D, E)** show the standardized mean difference (SMD) in CD56neg NK cell CD107a and IFNγ responses to HLA-negative target cells compared to CD56dim cells (CD107a REM SMD -0.74, CI ± 0.42, I2 = 54%, IFNγ REM SMD -1.29, CI ± 0.54, I2 = 73%; study n = 4, combined sample n = 182). Data are presented as mean and 95% CI.

We performed a meta-analysis of cell marker expression frequency, and the associated marker expression intensity, comparing CD56neg cells to the baseline set by CD56dim NK cells. **Figures 2A**, **B** show the REM results of data extracted from published studies and from CyTOF marker data combined (**Supplementary Table 3**). The NKG2, NKp30, NKp44, and NKp46 receptors all showed reduced expression frequency on CD56neg cells compared to the CD56dim NK subset, as did DNAM-1, the KIRs, LILRB1, 2B4, CD8, CD69, CD94, CXCR6, FcRg, CD161, NTB-A, Syk, Tactile, TNFα, CD57, Siglec-7, and perforin (**Figure 2A**). We included the CD57^+^NKG2C^+^ phenotype in our analysis which corresponds to the adaptive NK cell profile (22), finding a reduced frequency of CD56neg cells with this phenotype compared to CD56dim NK cells. In contrast, Granzyme B, CD2, CD38, CD62L, FAS-L, HLA-DR, Ki-67, PD-1, and TIGIT expression frequency was comparable between subsets. Regarding marker expression intensity, the CD56neg population showed significantly lower standardized expression of NKG2A, NKG2C, NKG2D, NKp44, KIR2DL1/S1, KIR2DL3, KIR2DL5, KIR2DS2, KIR2DS4, KIR3DL1, LILRB1, 2B4, CD2, CD62L, CD69, CD94, CXCR6, NTB-A, CD57, Siglec-7, and perforin compared to CD56dim NK cells (**Figure 2B**). The comparable expression of PD-1 and Ki-67 between the two NK cell subsets suggests that the CD56neg population does not contain a larger proportion of cells with an exhausted phenotype, nor reduced proliferation. The lower perforin and maintenance of FAS-L expression in the CD56neg NK cells could indicate a functional preference towards cell regulation *via* receptor-mediated suppression over cytotoxicity (78).

### CD56neg NK cell frequency increases with HIV-1 infection

We confirmed that HIV-1 infection correlates with an increased CD56neg subset frequency within NK cells. Loss of other NK subsets could be the cause. However, several studies that found increased CD56neg NK cell frequencies did not observe a corresponding reduction in total NK frequency within lymphocytes, and instead saw increased absolute counts of CD56neg NK cells (2, 4, 33, 45, 60, 65). This implies the CD56neg population is diluting the CD56^+^ populations either through proliferation, or phenotype switching of CD56^+^ cells to CD56neg. Compared to HIV-1^-^ donors, CD56neg NK cell frequencies in HIV-1^+^ individuals were expanded, showing an increase in mean of 10.69% over study controls (CI ± 3.34, I^2^ = 95%; **Figure 2C**). Cohorts of HIV-1^+^ virological controllers were included; long-term non-progressors (LTNP) who control HIV-1 in the absence of antiretroviral therapy (ART), and elite controllers (EC) who regulate HIV-1 without ART and show no immunological damage associated with HIV-1 infection. These individuals do not demonstrate the same increase in mean CD56neg NK cell frequency (50, 57, 58). This suggests that the immunological mechanism that promotes the subset’s expansion during chronic infection no longer acts when infection is controlled. While there are few studies investigating CD56neg NK cells in other chronic virus infections, our analysis of the available data (3, 4, 15, 19, 33, 35, 36, 45, 47, 48, 50–52, 54–58, 72, 79) indicates individuals with chronic viruses show an increase in mean of 10.05% (CI ± 3.11, I^2^ = 94%) in CD56neg NK cell frequency compared to controls (**Supplementary Figure 8**).

### CD56neg NK cells have reduced responses to HLA-negative targets compared to CD56dim cells

While several studies have reported that CD56neg NK cells have reduced responsiveness to HLA-negative target cells, variable methods were used. We combined the most widely used method, in which NK cells within PBMC were stimulated at a 5:1 effector-to-target ratio for a median of 6 hours, with HLA-negative target cells: K562 and 721.221 cells. We compared the CD107a and IFNγ expression of CD56neg cells to paired CD56dim responses (**Figures 2D, E**). Although CD56neg cells were responsive to HLA-negative targets, the combined standardized mean differences for both CD107a and IFNγ showed a reduction in response compared to CD56dim cells (-0.74, CI ± 0.42, I^2^ = 54% and -1.29, CI ± 0.54, I^2^ = 73% respectively).

### NK cell stimulation with IL-2 and IL-15 reduces the frequency of CD56neg NK cells

**Figure 3:**
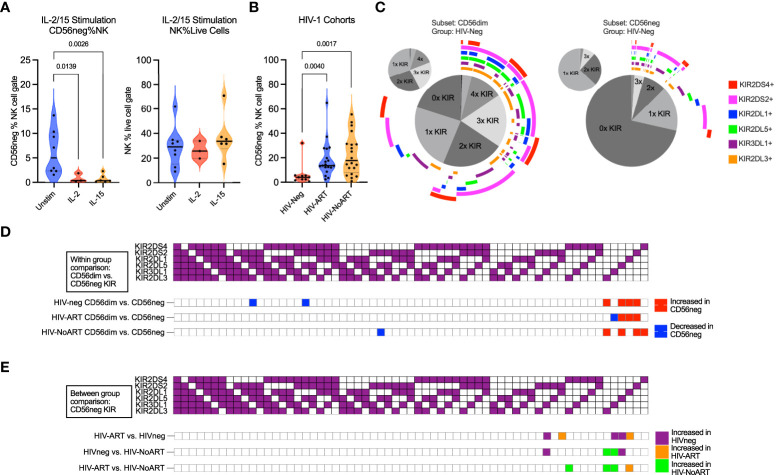
CD56neg subset frequency with IL-2 or IL-15 stimulation, frequency in HIV-1 dataset, and subset KIR repertoire comparison. **(A)** reduced CD56neg % NK when stimulated by IL-2 or IL-15 (FR-FCM-ZYZ3). CD56neg populations with under 100 events were excluded from analysis. **(B)** the frequency of the CD56neg subset within NK cells in HIV-1^-^ and HIV-1^+^ individuals on/not on ART (SDY1535). Kruskal-Wallis tests corrected for FDR were used for both analyses. **(C)** combinations of KIRs expressed by CD56dim and CD56neg cells in HIV-1^-^ group (SDY1535). The central pie chart shows proportion of subset expressing no KIR, one KIR, or combinations of KIRs. Surrounding bars denote the presence of KIRs corresponding to the legend color. The smaller orbiting pie charts show proportion of subset KIRs when KIR-negative cells were excluded. **(D)** comparison of KIR repertoire frequency between CD56dim and CD56neg subsets within each of the HIV-1^-^, HIV-1-ART, and HIV-1-NoART groups (SDY1535), with significantly larger (p < 0.05) KIR combination proportions on CD56neg (red) and CD56dim (blue) shown using two-way ANOVA. **(E)** impact of HIV-1 and ART status on KIR combination frequency within the CD56neg subset through between study group comparisons (HIV^-^ – purple, HIV-ART – orange, HIV-NoART – green).

NK cell survival, maturation, and proliferation can be promoted through culture in the presence of both IL-15 and IL-2 (80, 81). Additionally, IL-2 stimulation of sorted CD56neg NK cells from healthy donors promotes surface expression of CD56 following 5 days in culture (60). We used CyTOF datasets to analyze the response of CD56neg NK cells to cytokines, their KIR expression, their phenotypic heterogeneity, and their regulation by interaction with HLA class-I. We compared the proportion of CD56neg NK cells within total NK cells (CD3^-^CD14^-^CD19^-^CD7^+^ cells expressing CD56 and/or CD16) in the FR-FCM-ZYZ3 dataset between unstimulated cells, and cells stimulated either with IL-2 or IL-15 for 2.5 days (**Figure 3A**). Both cytokines decreased the proportion of CD56neg NK cells. As the NK proportion of live cells did not change, this suggests that a subset of the CD56neg NK cells are upregulating CD56 as described by Gonzalez et al. (60), as opposed to IL-2 and IL-15 stimulation specifically not promoting the proliferation of CD56neg NK cells.

### KIR expression and repertoire diversity are reduced in CD56neg NK cells compared to CD56dim NK cells

HIV-1 infection increases the frequency of CD56neg NK cells in the peripheral blood. Control of HIV-1 and the regulation of CD56neg subset frequency have both been linked to KIR expression (34, 37, 82, 83). We therefore sought to assess the difference in KIR repertoire between CD56neg cells and CD56dim cells, and between HIV-1^+^ and HIV-1^-^ groups. First, we confirmed that CD56neg cells had increased frequency in the HIV-1^+^ ART-treated and untreated groups compared to the HIV-1^-^ cohort in the SDY1535 CyTOF dataset (**Figure 3B**). Gates for positive KIR staining were set and Boolean gating was used to determine the presence of the possible KIR combinations in the CD56dim and CD56neg NK subsets. **Figure 3C** shows a plot visualizing the frequencies of possible KIR combinations among the KIR expressing cells. This plot emphasizes the higher proportion of CD56neg cells expressing no KIRs compared to CD56dim NK cells, as well as the larger proportion of CD56neg cells that express only one type of KIR. **Figure 3D** shows that in both the HIV-1^+^ and HIV-1^-^ groups, the KIR repertoire of the CD56neg cells is significantly different from that of the CD56dim subset, with the CD56neg population having a higher proportion of cells expressing a single KIR. Between group variation was similarly assessed (**Figure 3E**), finding that HIV-1 status, and treatment, influenced CD56neg NK cell KIR repertoire. HIV-1 status and treatment also impacted on KIR expression in CD56dim NK cells (**Supplementary Figure 9**). Functional analysis of CD56neg cells has shown a reduced responsiveness to target cells lacking HLA class-I. This could be explained by our observation of reduced KIR expression on these NK cells, and thus their lack of KIR-mediated education. Other possibilities include contamination of the CD56neg population by non-cytotoxic cells, or CD56neg cells containing subsets of different function.

### The perforin^-^CD94^-^NKG2C^-^NKp30^-^CD57^-^ subset of CD56neg cells accounts for differences in the CD56neg and CD56dim populations

**Figure 4:**
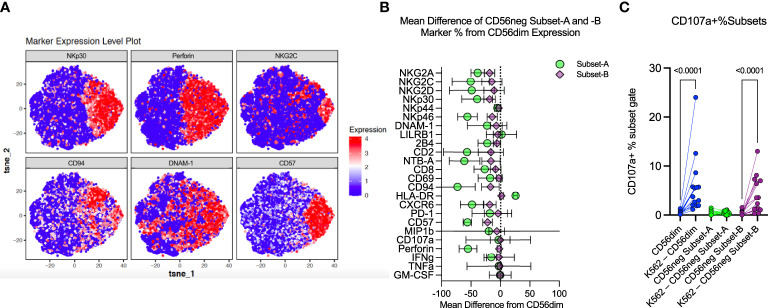
CD56neg cell analysis indicates distinct subset presence. **(A)** these tSNE plots were generated from exported CD56neg cells from the SDY517 dataset using the R cytofkit package, and show the main markers found to be significantly different in the Bozzano study that identified two subsets within the CD56neg NK cell population (32). Two subsets could be similarly defined through predominantly the expression of perforin, as well as surface expression of CD94 and other markers, to identify perforin^-^CD94^-^NKG2C^-^NKp30^-^CD57^-^DNAM-1^-^ (Subset-A) cells, and all other CD56neg NK cells (Subset-B). **(B)** this plot shows the combined mean difference of Subset-A and -B CD56neg cell frequency marker expression from CD56dim NK cells from the datasets SDY517, SDY1535, and FR-FCM-ZYZ3. **(C)** this graph shows the change in CD107a^+^ CD56dim, Subset-A and -B CD56neg NK cell frequencies, comparing unstimulated expression to K562 stimulated levels (SDY517).

Bozzano et al. have indicated that the CD56neg NK population has its own subsets defined by expression of perforin, CD94, NKG2C, NKp30, CD57 and DNAM-1 (16). We exported the CD56neg population from the SDY517 CyTOF dataset and ran a tSNE dimensionality reduction on these cells. As shown in **Figure 4A**, the CD56neg cells comprise two subpopulations, distinguished by their expression of perforin and CD94. By analyzing the SDY517, SDY1535, and FR-FCM-ZYZ3 datasets we defined a perforin^-^CD94^-^NKG2C^-^NKp30^-^CD57^-^ subset of CD56neg NK cells (Subset-A). We then used Boolean gating to define the remaining CD56neg subset of NK cells that lacks the Subset-A phenotype and expresses one or more of the perforin, CD57, NKG2C, NKp30 and CD94 markers (Subset-B) (**Supplementary Figures 1–3**). Through REM comparison of marker expression frequency between CD56neg cell subsets and the CD56dim NK cells, we found the CD56neg Subset-B cells are phenotypically closer to the CD56dim cells than to Subset-A cells (**Figure 4B**). Expression frequency of NKG2A, NKp30, NKp46, CD94, CD57, NTB-A, and CXCR6 was significantly lower in the CD56neg Subset-B population than in the CD56dim cells, thus resembling a less mature CD56dim NK phenotype (20). While expression frequency of NKG2A, NKG2D, NKp44, NKp46, 2B4, CD2, CD8, NTB-A, CXCR6, and IFNγ was lower on the CD56neg Subset-A cells than CD56dim cells, they had higher HLA-DR expression frequency than either the CD56neg Subset-B or CD56dim populations. To confirm that the larger proportion of the HLA-DR^+^ CD56neg Subset-A cells are NK cells and not dendritic cells (T and B-cells being already excluded through gating), we compared their KIR expression to that of HLA-DR^-^ CD56neg Subset-A cells. Some KIR were more highly expressed in HLA-DR^+^ cells and others were more highly expressed in the HLA-DR^-^ subset. Thus there was no consistent variation in KIR expression related to HLA-DR expression in the CD56neg Subset-A NK cells (**Supplementary Figure 10**). Overall, these results suggest that much of the phenotypic variation distinguishing CD56neg and CD56dim NK cells is due to the CD56neg Subset-A population, which could also include a population of NK cell progenitors (16).

### CD56neg Subset-B NK cells and CD56dim NK cells respond similarly to HLA class-I-deficient target cells

**Figure 5:**
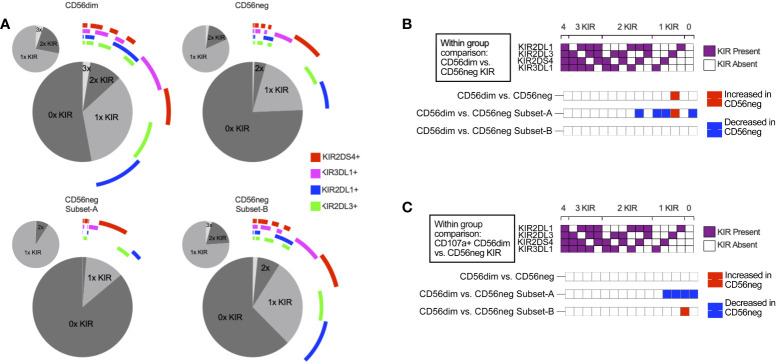
KIR repertoire comparison of CD56neg NK subsets. **(A)** these charts show the combinations of KIRs being expressed by CD56dim cells, CD56neg cells, and CD56neg Subset-A and -B from the SDY517 Immport.org dataset. The central pie chart with the encircling bars shows the proportion of either the CD56dim or CD56neg cell subsets that express no KIR, a single KIR, or combinations of KIRs. The bars around the plot denote the presence of a specific KIR corresponding to the legend color, and the smaller pie charts orbiting the central plot show the proportion of subset KIRs when KIR-negative cells were excluded. **(B)** this heatmap shows a comparison of KIR repertoire frequency between CD56dim and the CD56neg, Subset-A and -B cells (SDY517). **(C)** this heatmap shows cells that express CD107a^+^ following K562 stimulation, comparing the KIR repertoire of CD107a^+^ CD56dim to CD56neg cells, and CD56neg Subset-A and -B (SDY517). Significantly varying KIR repertoire frequency between subsets (p < 0.05) are shown as red based on two-way ANOVA results with multiple comparisons corrected using the Sídák method.

NK cells challenged with the HLA class-I-deficient K562 cells were also included in the SDY517 dataset. CD56dim cells and the small CD56neg Subset-B population exhibited comparable increases in CD107a expression following coculture with K562 cells. In contrast, no increase in CD107a expression was observed for CD56neg Subset-A cells (**Figure 4C**). Direct unpaired comparison of K562 stimulated subsets using an FDR corrected Kruskal-Wallis test found that CD56neg Subset-A cells express significantly less CD107a than the CD56dim cells (q-value < 0.0001), whereas Subset-B cells did not vary significantly (q-value = 0.0905). Thus CD56neg Subset-B NK cells respond to HLA-negative target cells as well as the CD56dim cells. The IFNγ, TNFα, IL-10, IL-17a, and GM-CSF responses did not significantly change on stimulation with K562 for any of the NK cell subsets (**Supplementary Figure 11**). To determine if KIR-mediated NK cell education contributed to differences in the “missing-self” response, we compared the KIR repertoire of CD56dim, CD56neg Subset-A, and Subset-B NK cells (**Figure 5A**). No significant differences in KIR repertoire were observed between CD56dim and CD56neg Subset-B NK cell responses (**Figure 5B**). In contrast to CD56dim cells, Subset-A NK cells demonstrated a decreased proportion of cells expressing a single KIR type. Furthermore, NK cells expressing KIR2DL1 as their only KIR were at higher frequency in CD107a^+^ CD56neg Subset-B NK cells than in CD107a^+^ CD56dim cells (**Figure 5C**), whereas CD107a^+^ CD56neg Subset-A cells had fewer cells expressing a single KIR than the CD56dim population. Similarity in KIR expression and response to K562 targets by the CD56dim and CD56neg Subset-B cells indicates these subsets are similarly regulated by KIR-mediated NK cell education.

### Gene expression signatures associated with Subset-A and -B CD56neg NK cells

**Figure 6:**
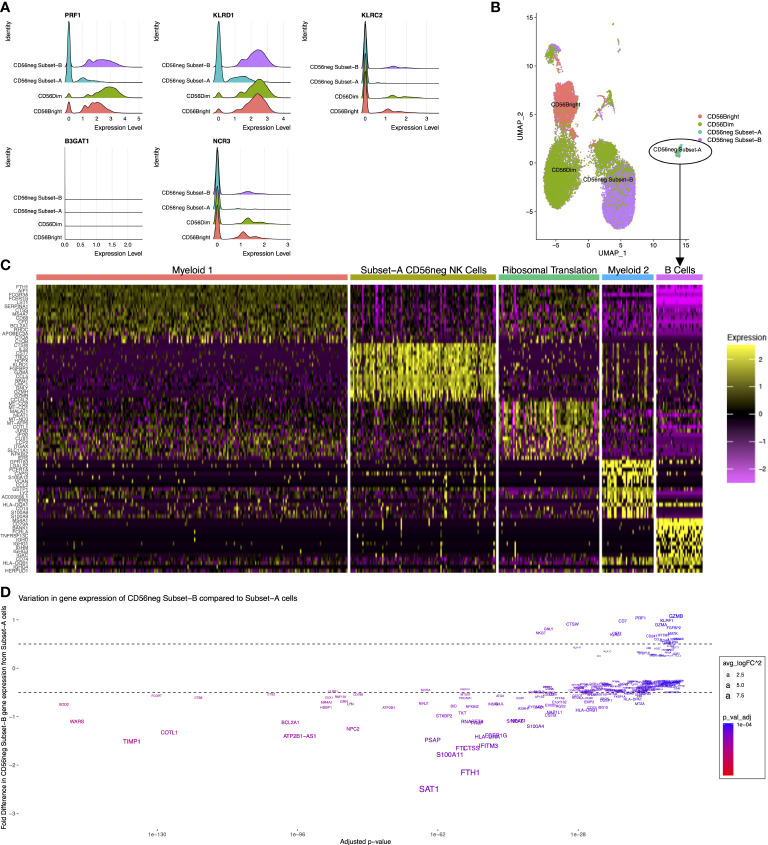
Distinct CD56neg subset gene expression. These graphs show the CD56neg cell population from the Bradley 2018 scRNAseq dataset on NK cells from an HIV-1^+^ donor (24). **(A)** histograms of NK cell subset gene expression of perforin (*PRF1*), CD94 (*KLRD1*), NKG2C (*KLRC2*), CD57 (*B3GAT1*), and NKp30 (*NCR3*). **(B)** UMAP of CD56bright, CD56dim, and CD56neg meta-clusters. **(C)** top fifteen differentially expressed genes for the 5 clusters identified within CD56neg Subset-A. One cluster demonstrated NK cell gene expression including *CD7*, *NKG7*, *GNLY*, and granzyme genes. Other clusters had myeloid, B-cell, or ribosome-associated transcriptomes. **(D)** variation in gene expression of Subset-B CD56neg cells compared to the NK cell cluster within the Subset-A CD56neg subset. Adjusted p-values for differential gene expression level are shown on the x-axis, and the fold difference in expression of Subset-B cells to Subset-A on the y-axis. Gene name sizes correspond to their fold difference in expression, and the level of significant difference between subsets is shown by increasing red coloration.

CyTOF dataset analysis showed there are phenotypic and functional differences between the CD56neg Subset-A and Subset-B NK cells. We therefore examined if gene expression differences in the CD56neg population defines similar subsets, using the scRNAseq dataset from Bradley et al. (33) where CD56bright, CD56dim, and CD56neg NK cells were sorted prior to sequencing. We performed meta-clustering of unbiased CD56neg cell clusters that resulted in the generation of a perforin^-^CD94^-^NKG2C^-^NKp30^-^ population (*PRF1^-^KLRD1^-^KLRC2^-^NCR3^-^*) corresponding to CD56neg Subset-A, and a population corresponding to Subset-B, which we then incorporated with paired scRNAseq data for CD56bright and CD56dim cells (**Figure 6A**). CD57 (*B3GAT1*) expression which we previously used in cytometry data for Subset-A and -B definition was not observed in any NK cell subset (**Figure 6A**). The CD56neg Subset-B cells closely associated with a CD56dim UMAP population, whereas the Subset-A NK cells clustered separately (**Figure 6B**).

To assess if the Subset-A meta-cluster consisted of only NK cells we assessed the differentially expressed genes within the CD56neg Subset-A meta-cluster (**Figure 6C**). Five clusters were identified by differential gene expression analysis, and informed by g:Profiler. One of them, called the Subset-A CD56neg NK Cell Cluster, was defined by expression of *CD7*, *NKG7*, *GNLY*, and granzyme. Two clusters expressed myeloid cell-associated genes, one expressed B-cell genes, and the remaining cluster demonstrated expression related to ribosomal translation without a distinctive immune cell profile (Supplementary File 3). This adds transcriptional evidence for the presence of a perforin^-^CD94^-^NKG2C^-^NKp30^-^ CD56neg subset of NK cells.

We compared gene expression between the Subset-A CD56neg NK Cell Cluster and CD56neg Subset-B NK cells (**Figure 6D**). Gene expression associated with CD56neg Subset-B NK cells included *NKG7*, *GNLY*, *CTSW*, *HLA-A*, *B2M*, *HLA-C*, *KLRD1*, *CST7*, *CD7*, *RPL3*, *PRF1*, *RPS3*, *RPL31*, *UBB*, *RPL23A*, *CD247*, *RPL37*, *RPS27A*, *RPS26*, and *HCST*, whereas the CD56neg Subset-A population had greater expression of *CPPED1*, *SIGLEC10*, *LY96*, *NCF2*, *CYBB*, *PHACTR1*, *C19orf38*, *HCK*, *MNDA*, *SLC7A7*, *LILRA1*, *MARCKS*, *TCF7L2*, *C5AR1*, *CFP*, *CTSL*, *CDKN1A*, *SLC11A1*, *PILRA*, and *KLF4*. Significantly, more highly expressed genes in CD56neg Subset-B cells were associated with ribosome translation and protein trafficking pathways, as well as viral gene expression and translation, while the more highly expressed genes in CD56neg Subset-A cells were associated with pathways of cell activation and innate immune responses (Supplementary File 3). This suggests that the two CD56neg NK subsets have varying transcriptional responses to the donor’s ongoing HIV-1 infection.

The Bozzano et al. study (16) suggested the Subset-A cells were progenitor cells, so we investigated gene expression differences associated with NK cell development (**Supplementary Figure 12**); *NOTCH1* expression is associated with common lymphoid progenitor cells, *ID2* and *RUNX* expression with NK progenitor cell differentiation, *E4BP4* (*NFIL3*) and *ETS1* expression mark the development of NK progenitors to immature NK cells, and the presence of *EOMES* is linked to developing NK cells that then gain *T-bet* (*TBX21*) as they mature (84). Previous studies show that CD56neg NK cells express similar levels of *EOMES* and *T-bet* as CD56dim cells (35, 85). Each defined NK subset showed expression of these genes, and although expression of *ID2*, *RUNX3*, and *ETS1* were lower in the CD56neg Subset-A than Subset-B cells, differential gene analysis showed it was not a significant difference. With the limited cell number and their transcriptional variation, we cannot conclude if this subset contains progenitor cells.

### CD56neg subset frequency is associated with HLA-E expression, whereas CD56neg Subset-B proportion associates with HLA-C expression

**Figure 7:**
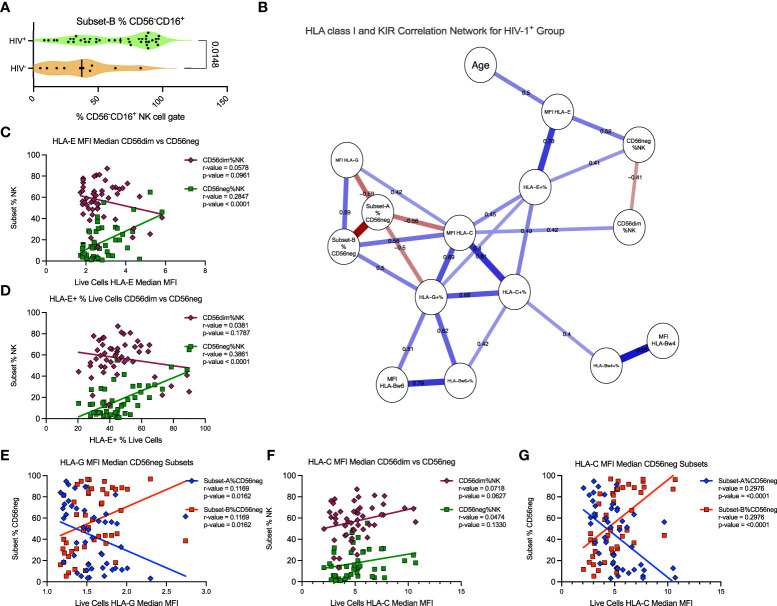
HLA-C expression associates with Subset-B frequency within CD56neg NK cells in HIV-1^+^ donors. **(A)** frequency of the Subset-B population within CD56neg NK cells, comparing HIV^-^ and HIV-1^+^ donors (SDY1535) using two-tailed Mann-Whitney analysis. Median and IQR shown. **(B)** Spearman’s correlation network corrected using the FDR method, with significant (p < 0.05) relationships between the frequency and expression intensity of leukocyte HLA class-I molecules and the frequency of CD56neg and CD56dim subset within NK cells, and of Subset-A and -B frequencies within CD56neg cells of HIV-1^+^ donors (SDY1535). Blue lines show positive associations while red lines show inverse correlations, with correlation coefficients included above the lines. The plot was generated using qgraph (36). **(C, D)** show linear regression of CD56dim (maroon diamonds) and CD56neg (green squares) frequencies within NK cells to **(C)** HLA-E expression intensity on live cells, and **(D)** HLA-E expression frequency. **(E)** shows linear regression of Subset-A (blue diamonds) and Subset-B (red squares) frequencies within CD56neg NK cell gate with the median HLA-G expression intensity for the live cells from the same samples. **(F, G)**. show linear regression analysis of with HLA-C expression intensity on live cells, of **(F)** CD56dim and CD56neg cells, and **(G)** CD56neg Subset-A and -B frequencies.

Further analysis of the SDY1535 dataset showed that Subset-B NK cells comprise a significantly larger proportion of the CD56neg cells in HIV-1^+^ donors (**Figure 7A**). This suggests that the expansion of the CD56neg subset during HIV-1 infection could be a consequence of positive selection for the Subset-B population. Using matched dataset samples with HLA class-I staining we exported the HLA expression data for total live cells, and generated a correlation network of the HIV-1^+^ donors. With this network we explored the potential influence of HLA class-I expression and donor age on the expansion of the CD56neg subset of NK cells, and on the proportion of Subset-A and -B CD56neg cells (**Figure 7B**). Both the frequency of PBMC expressing HLA-E, and the intensity of HLA-E surface expression, were positively associated with CD56neg cell frequency within the NK population (**Figures 7B–D**). Age did not correlate with CD56neg cells, nor with Subset-A or -B frequencies, but did correlate positively with HLA-E expression intensity (**Figure 7B**). The frequency and expression intensity of HLA-G were inversely correlated with the frequency of Subset-A cells within the CD56neg NK cell population (**Figures 7B, E**). HLA-C expression has been previously associated with the CD56neg cell subset frequency (83), although our result lacked statistical significance (**Figure 7F**). However, our regression analysis implicates HLA-C as a promotor of the Subset-B NK cells (**Figure 7G**).

## Discussion

We set out to address outstanding questions pertaining to the subset of human NK cells that lack CD56 expression (CD56neg cells). Combining meta-analysis of published investigations and deposited CyTOF datasets, we demonstrate that CD56neg cells comprise a stable population of peripheral blood NK cells. To a varying degree the CD56neg cells are also present in tissues. Furthermore, they divide into a perforin^-^CD94^-^NKG2C^-^NKp30^-^CD57^-^ subset (Subset-A) and a subset consisting of all other CD56neg cells (Subset-B), and these two cell groups have distinct phenotypic and transcriptomic signatures. The KIR repertoire, functional response against HLA-lacking targets, and regulation by HLA class-I expression were all determined to vary between the two CD56neg NK cell subpopulations. Subset-A was the least like classical CD56dim NK cells, whereas Subset-B more closely resembles CD56 expressing NK cells and constitutes a greater proportion of CD56neg NK cells in HIV-1^+^ individuals.

While this work sheds light on the CD56neg NK cells, key questions remain unanswered. Do the CD56neg NK cells have a common ontogeny with the CD56dim NK cells? Are CD56neg Subset-A cells a precursor for CD56 positive NK cells (16), and does their higher expression of HLA-DR confer a unique function? What is the role of CD56neg cells in chronic viral infections, and what is the mechanism expanding their population? Do they restrict viraemia or slow immune exhaustion and/or damage? Are CD56neg cells promoted in other chronic infections, such as HTLV or syphilis? What cells are the CD56neg subsets interacting with in blood and tissues? How do they affect immune function? And how do human CD56neg cells compare to non-human primate NK cells in development and function?

During NK cell development, CD56bright and CD56dim cells are considered to originate from a common innate lymphoid progenitor that also gives rise to various classes of innate lymphoid cells (ILCs) (86). From the NK cell progenitor, it is then possible to define linear stages of NK development using cell surface molecules that ends with mature CD56dim CD16^+^CD57^+^KIR^+^NKp80^+^ cells (20). However, NK cells are a highly diverse population (21) and the influence of pathogens and immune factors can promote variation in development (22). We found that the CD56neg Subset-B cells are phenotypically close to CD56dim cells. If we assume the linear development from CD56bright cells into CD56dim NK cells, then Subset-B corresponds to the mature CD56dim population that expresses CD16, has decreased NKG2A expression and has yet to express CD57 (20). However, CD56neg Subset-B cell KIR expression reflects the KIR expression of CD56dim NK cells. KIR expression is considered stochastic (87), implying that the CD56neg cells have a similar range of maturation states as the CD56dim subset. This similarity between the subsets suggests either that Subset-B CD56neg cells develop separately from CD56^+^ NK cells but are subject to similar influences, leading to similar maturation and KIR expression, or that CD56 expression can be lost or gained and the CD56dim and CD56neg populations have a common ontogeny.

A proteomic comparison of CD56dim and CD56neg NK cell subsets demonstrated that they were phenotypically homogenous, with the only differences being that CD56dim cells had increased FcRL6 and CD319 expression, while CD56neg cells had higher levels of surface CD127 and CD172γ (85). Absence of CD56 and presence of CD127 is associated with an ILC phenotype. However, ILCs do not express CD16 or KIRs, nor do they produce perforin or degranulate in the presence of K562 targets (86). The case for CD56neg Subset-A cells is uncertain, due to their reduced KIR expression and lack of response to K562 cells. Bozzano et al. characterized this CD56neg perforin^-^CD94^-^NKG2C^-^NKp30^-^CD57^-^ population as an intermediate between CD34^+^DNAM-1^bright^CXCR4^+^ lymphoid progenitors that can give rise to mature NKG2C^+^ NK cells that are functionally effective and potent regulators of CMV replication (16). However, they did not conclude whether the other CD56neg cells were also produced by the same progenitor population (16). This suggests CD56neg Subset-A cells are an alternative progenitor to NK cells, specifically related to ongoing CMV infection if not other chronic virus infections. The transcriptomic profile of CD56neg NK cells has been described as similar to that of CMV associated adaptive CD56dim cells, further supporting this theory (47).

If we look to gene expression for guidance, CD56neg Subset-B cells expressed similar levels of development associated genes to the CD56dim subset, which with their similarity in KIR expression and functional response towards K562 cells, supports these cells being a mature NK population. While not significant, CD56neg Subset-A cells expressed lower levels of *ID2*, *RUNX3*, and *ETS1* in comparison to CD56neg Subset-B cells and other NK populations, suggesting they could be in an earlier stage of development (84). This fits with the notion of them being a progenitor population.

Direct comparison of Subset-A and -B cells using scRNAseq indicated that the populations have distinct gene expression profiles. Subset-A demonstrated variation from other NK cell subsets as evidenced by the grouping of the Subset-A CD56neg NK Cell Cluster with myeloid and B cell clusters, being distinguished from these non-NK cells by the expression of *CD7*, *NKG7*, and *GNLY*. The expression of *APOBEC3A* and *HLA-DQB1* in the two myeloid populations indicates that a small number of monocytes and/or dendritic cells were not excluded by the CD3^-^CD14^-^CD19^-^ flow cytometry cell sorting. The expression of *CD79A* and immunoglobulin genes similarly suggests the presence of B cells. The Subset-A cells were most likely clustered with the myeloid and B cell populations due to their distinction from other NK subsets instead of being more related to these non-NK cells. If Subset-A is a progenitor population it may contain gene expression homologous to lymphocyte and myeloid subsets that would group it with monocytes, dendritic cells, and B cells. However, our investigation could not confirm that Subset-A cells were a progenitor population.

The development of CD56neg NK cells is clearly affected by chronic viral infections. The most studied, HIV-1, demonstrates significant expansion of the CD56neg proportion in NK cells. We found that this expansion is associated with CD56neg Subset-B, indicating that HIV-1 infection promotes this population’s generation either through expansion or through loss of CD56 expression from CD56dim cells. It has been suggested that the change in CD56neg frequency in NK cells in chronic infection is due to loss of CD56dim NK cells instead of CD56neg expansion. However, a number of studies have demonstrated growth in the CD56neg NK cell population while NK frequency within lymphocytes remains stable, or directly through increased CD56neg absolute cell counts (2, 4, 33, 45, 60, 65).

Altered cytokine signaling during chronic infections may promote this NK cell phenotype, as a case study of a Janus kinase-3 deficient donor with altered cytokine signaling capacity identified an expanded population of CD3^-^CD56^-^CD16^+^KIR^+^ cells (88). Changes in HLA class-I expression likely play a role as well. Increased HLA-C surface expression has been found to negatively correlate with CD56neg cell frequency in NK cells in HIV-1^+^ individuals, and that this was exaggerated by HLA-C1^+^/C1^+^ KIR2DL3^+^KIR2DL2^-^ genotypes, suggesting that while chronic infection expands this population there is a genetic mechanism also regulating CD56neg NK cell development (83). This implies that HLA-C2^+^ education of NK cells may preferentially expand the CD56neg population, which is supported by the greater proportion of CD56neg and CD56dim cells expressing KIR2DL1 alone that we observed with HIV-1^+^ status in the SDY1535 dataset. Our analysis found a positive but non-significant relationship between HLA-C expression level and CD56dim frequency in NK cells. However, within CD56neg cells, Subset-B frequency positively correlated with both HLA-C and HLA-G expression levels, supporting the notion that interactions between HLA-C and KIRs regulate these NK cells. The increased HLA-C expression observed on CD3^-^CD19^-^CD33^-^CD56^-^CD16^+^ cells associated with CMV reactivation may even be a method of self-promotion (89).

HLA-E expression positively correlated with CD56neg frequency in NK cells. Compared to CD56dim cells, CD56neg NK cells showed minor variation in NKG2C and reduced NKG2A expression, indicating that their association with HLA-E reflects a reduced capacity for HLA-E:NKG2A inhibition. This mechanism is similar to the greater response to HLA-E expressing CMV infected endothelial cells found in NKG2C^+^ versus NKG2A^+^ CD56dim cells (90). HIV-1 reportedly stabilizes HLA-E surface expression to reduce NK cell activation and reduce lysis of infected CD4 T cells (91). HLA-E maintenance in CMV infected cells has also been demonstrated (92), suggesting that at least the CD56neg Subset-B expansion is an adaptation to this immune evasion strategy. Due to its reduced NKG2A expression, it is likely that the CD56neg Subset-B population is more capable of lysing virally infected cells expressing increased levels of HLA-E.

Humans seem to have gained greater expression of CD56 on NK cells than our primate cousins (23–25, 28, 29). We hypothesize that this gain of CD56, in addition to the changes in KIR expression and expansion of NK subsets, such as adaptive or CD56neg cells observed in HIV-1, HCV, EBV and CMV, were driven by historical chronic infections that acted as selective pressures on human evolution, driving the genetic diversity of HLA and KIRs in our species. In evolutionary terms, the emergence of HIV-1 and HCV infections is relatively recent (~100 and ~3000 years) (93, 94), whereas CMV and EBV have an intimate relationship with primates measured in millions of years (95–97). Consequently, these herpesviruses have had a greater influence on the evolution of the immune system. Their association with adaptive NK cells and CD56neg NK subsets implies these diverse innate lymphocytes represent biological adaptations to control or limit the pathogenicity of chronic viral infections.

## Data availability statement

The original contributions presented in the study are included in the article/**Supplementary Material**. Further inquiries can be directed to the corresponding author.

## Author contributions

Conceptualization: AC, ZD, PP. Methodology: AC, LG. Investigation: AC. Writing: AC, FL, ZD, LG, PP. All authors contributed to the article and approved the submitted version.

## Funding

This work was generously funded by a National Institutes of Health grant R01 AI136952, and a Stanford School of Medicine Discovery Innovation Award.

## Acknowledgments

We thank the past and present members of the Parham laboratory who provided critical feedback during the course of this work. Additionally, we thank the members of the research groups who made their data publicly available for further analysis, for the ongoing work of the repositories in which these datasets are maintained, as well as for all the people running and participating in the research that we have included in this paper.

## Conflict of interest

The authors declare that the research was conducted in the absence of any commercial or financial relationships that could be construed as a potential conflict of interest.

## Publisher’s note

All claims expressed in this article are solely those of the authors and do not necessarily represent those of their affiliated organizations, or those of the publisher, the editors and the reviewers. Any product that may be evaluated in this article, or claim that may be made by its manufacturer, is not guaranteed or endorsed by the publisher.
