# Supplementary material for: CD56-negative NK cells: Frequency in peripheral blood, expansion during HIV-1 infection, functional capacity, and KIR expression
Source: Front Immunol. 2022 Sep 23;13:992723. doi: 10.3389/fimmu.2022.992723 (PMC9539804; doi:10.3389/fimmu.2022.992723)
Supplement: Supplementary file 1 [file DataSheet_1.pdf]

Supplementary Table 1 – MOOSE Reporting Criteria Table

The table below shows our reporting checklist based on the proposed "Meta-analysis of Observational Studies in Epidemiology" (MOOSE) guidelines, with the relevant sections of this paper reported (Stroup et al., 2000)

Item No	Recommendation	Reported
	Reporting of background	
1	Problem definition	Yes: Introduction
2	Hypothesis statement	Yes: Introduction
3	Description of study outcome(s)	Addressed as aims in Introduction
4	Type of exposure or intervention used	Not applicable
5	Type of study designs used	Observational studies
6	Study population	Studies of humans only
	Reporting of search strategy	
7	Qualifications of searchers (eg, librarians and investigators)	Completed by study investigators
8	Search strategy, including time period included in the synthesis and key words	Search strategy included in Methods
9	Effort to include all available studies, including contact with authors	Only published data was collected
10	Databases and registries searched	Results
11	Search software used, name and version, including special features used (eg, explosion)	No search software package was used
12	Use of hand searching (eg, reference lists of obtained articles)	Undertaken by investigators
13	List of citations located and those excluded, including justification	Supplementary File 4
14	Method of addressing articles published in languages other than English	None were returned in the search, however where no published translation exists they were to be excluded
15	Method of handling abstracts and unpublished studies	Excluded
16	Description of any contact with authors	None. Required data was extracted directly from paper text and graphs; Results
	Reporting of methods	
17	Description of relevance or appropriateness of studies assembled for assessing the hypothesis to be tested	Strict definition for CD56neg NK subset was used; Results
18	Rationale for the selection and coding of data (eg, sound clinical principles or convenience)	Not applicable
19	Documentation of how data were classified and coded (eg, multiple raters, blinding and interrater reliability)	Not applicable
20	Assessment of confounding (eg, comparability of cases and controls in studies where appropriate)	Not applicable
21	Assessment of study quality, including blinding of quality assessors, stratification or regression on possible predictors of study results	Not applicable
22	Assessment of heterogeneity	High heterogeneity (I^2=50-97%) across studies, which was expected as we assessed observational data from varied cohorts
23	Description of statistical methods (eg, complete description of fixed or random effects models, justification of whether the chosen models account for predictors of study results, dose-response models, or cumulative meta-analysis) in sufficient detail to be replicated	Detailed in Methods
24	Provision of appropriate tables and graphics	Yes; Figure 1, Supplementary Table 1-3,
	Reporting of results	
25	Graphic summarizing individual study estimates and overall estimate	Figure 1
26	Table giving descriptive information for each study included	Figure 1
27	Results of sensitivity testing (eg, subgroup analysis)	Not applicable
28	Indication of statistical uncertainty of findings	Yes; Results
	Reporting of discussion	
29	Quantitative assessment of bias (eg, publication bias)	Not done
30	Assessment of quality of included studies	All studies included met our requirement for the definition of CD56neg NK cells
	Reporting of conclusions	
31	Consideration of alternative explanations for observed results	We consider the possible effect of contaminating cell populations in the Discussion
32	Generalisation of the conclusions	We discuss our results in the context of general NK cell development as well as in chronic infection settings
33	Guidelines for future research	Yes
34	Disclosure of funding source	Yes

D. F. Stroup *et al.*, Meta-analysis of observational studies in epidemiology - A proposal for reporting. *Jama-Journal of the American Medical Association* **283**, 2008-2012 (2000)

Supplementary Table 2 – REM analysis with median/IQR data excluded

The table below shows the results of our REM analyses where we included median and converted IQR data, and the results when only mean and SD data was used.

REM Analysis	REM including median/IQR	REM % ±CI	Combined sample n	Publication n/ CyTOF n	REM excluding median/IQR	REM % ±CI	Combined sample n	Publication n/ CyTOF n
CD56neg%NK Blood Paper + CyTOF combined	5.67	1.22	757	28/6	*5.93*	*1.57*	*437*	*14/6*
CD56neg%NK Blood Paper Only	5.65	1.21	653	28/0	*5.93*	*1.53*	*333*	*14/0*
CD56neg%NK Blood CyTOF Only	6.11	5.92	104	0/6	*6.11*	*5.92*	*104*	*0/6*
CD56neg%NK Non-Blood	12.38	5.32	96	2/2	*12.39*	*5.78*	*86*	*1/2*
HIV-1 Mean Difference in CD56neg%NK	10.69	3.34	751 vs 354	15/1	*12.49*	*5.57*	*199 vs 138*	*6/1*
CD107a Standardized Mean Difference CD56neg vs CD56dim	-0.74	0.42	182	4/0	*-0.84*	*0.78*	*14*	*1/0*
IFNγ Standardized Mean Difference CD56neg vs CD56dim	-1.29	0.54	182	4/0	*-1.00*	*0.79*	*14*	*1/0*

Supplementary Table 3 – REM results with and without median/IQR data included

The table below shows the results of our REM analyses where we both included and excluded median and converted IQR data. Results in bold text indicate where exclusion of median and converted IQR data alters the analysis findings.

Marker	REM mean difference CD56neg from CD56dim	REM % ±CI	Combined sample n	Publication n/ CyTOF n	REM mean difference CD56neg from CD56dim excluding median/IQR data	REM % ±CI	Combined sample n	Publication n/ CyTOF n
Marker mean %								
NKG2A	-14.90	3.58	356	6/3	-16.76	4.47	214	4/3
NKG2C	-13.87	4.36	565	6/3	-16.72	5.66	256	3/3
NKG2D	-11.17	3.94	221	3/3	-11.17	3.94	221	3/3
NKp30	-23.46	6.31	315	6/2	-25.02	8.07	206	4/2
NKp44	-13.78	6.51	104	0/3	-13.78	6.51	104	0/3
NKp46	-21.11	9.11	202	5/1	-24.50	10.37	172	4/1
Perforin	-12.70	6.70	66	1/2	-12.70	6.70	66	1/2
Granzyme B	-5.99	9.61	48	2/1	**-16.14**	**4.38**	**28**	**1/1**
Siglec-7	-15.01	4.47	253	4/2	-13.92	5.73	160	2/2
DNAM-1	-7.51	4.19	104	0/3	-7.51	4.19	104	0/3
KIR2DL1/S1	-15.48	3.32	233	2/3	-15.48	3.32	233	2/3
KIR2DL3	-15.30	5.56	183	1/3	-17.13	6.05	104	0/3
KIR2DL5	-19.65	6.09	104	0/3	-19.65	6.09	104	0/3
KIR2DS2	-15.81	5.88	87	0/2	-15.81	5.88	87	0/2
KIR2DS4	-10.73	4.83	104	0/3	-10.73	4.83	104	0/3
KIR3DL1	-14.93	5.69	152	1/3	-14.93	5.69	152	1/3
LILRB1	-9.97	5.16	167	1/3	-13.30	4.49	104	0/3
2B4	-8.39	4.13	104	0/3	-8.39	4.13	104	0/3
CD2	-9.97	11.31	60	1/2	-9.97	11.31	60	1/2
CD8	-9.78	3.83	162	2/3	-9.78	3.83	162	2/3
CD38	-3.91	8.49	135	1/2	-3.91	8.49	135	1/2
CD62L	1.03	2.19	173	3/2	1.03	2.19	173	3/2
CD69	-3.89	3.40	229	3/3	-3.89	3.40	229	3/3
CD94	-16.60	7.86	171	2/2	-12.67	6.84	92	1/2
CXCR6	-23.42	5.14	104	0/3	-23.42	5.14	104	0/3
FAS-L	-6.93	10.61	87	0/2	-6.93	10.61	87	0/2
FcRg	-7.64	4.78	150	1/2	-10.05	6.41	87	0/2
HLA-DR	-2.91	7.32	135	1/2	-2.91	7.32	135	1/2
Ki-67	-3.99	4.75	107	1/2	-3.99	4.75	107	1/2
NTB-A	-14.62	8.67	87	0/2	-14.62	8.67	87	0/2
PD-1	-2.08	7.68	104	1/2	-2.08	7.68	104	1/2
Syk	-7.69	5.86	87	0/2	-7.69	5.86	87	0/2
Tactile	-16.89	7.76	49	0/1	-16.89	7.76	49	0/1
TIGIT	-8.06	9.94	87	0/2	-8.06	9.94	87	0/2
CD57	-28.95	3.64	392	6/3	-28.87	4.15	329	5/3
$CD57^{+}NKG2C^{+}$	-29.27	9.03	87	0/2	-29.27	9.03	87	0/2
CD161	-25.49	18.99	182	3/1	**-25.86**	**26.35**	**103**	**2/1**
TNFa	-6.38	3.58	80	1/1	-6.68	5.53	17	0/1
Marker mean MFI								
NKG2A	-0.71	0.51	132	1/3	-0.96	0.26	104	0/3
NKG2C	-0.79	0.40	132	1/3	-0.94	0.35	104	0/3
NKG2D	-0.57	0.45	132	1/3	-0.35	0.21	104	0/3
NKp30	-0.36	0.40	85	1/2	**-0.58**	**0.40**	**55**	**0/2**
NKp44	-1.07	0.52	104	0/3	-1.07	0.52	104	0/3
NKp46	-0.24	0.70	75	2/1	0.35	1.11	17	0/1
Perforin	-0.51	0.50	104	0/3	-0.51	0.50	104	0/3
Granzyme B	0.05	0.13	37	1/1	0.01	0.22	17	0/1
Siglec-7	-1.31	0.38	117	1/2	-1.58	0.39	87	0/2
DNAM-1	-0.71	0.87	104	0/3	-0.71	0.87	104	0/3
KIR2DL1/S1	-0.46	0.37	185	1/3	-0.46	0.37	185	1/3
KIR2DL3	-0.62	0.47	104	0/3	-0.62	0.47	104	0/3
KIR2DL5	-1.02	0.37	104	0/3	-1.02	0.37	104	0/3
KIR2DS2	-1.69	0.52	87	0/2	-1.69	0.52	87	0/2
KIR2DS4	-0.28	0.19	104	0/3	-0.28	0.19	104	0/3
KIR3DL1	-0.92	0.21	104	0/3	-0.92	0.21	104	0/3
LILRB1	-0.95	0.27	104	0/3	-0.95	0.27	104	0/3
2B4	-0.47	0.29	104	0/3	-0.47	0.29	104	0/3
CD2	-0.57	0.45	60	1/2	-0.57	0.45	60	1/2
CD8	-0.26	0.29	114	1/3	-0.26	0.29	114	1/3
CD38	0.42	0.66	87	0/2	0.42	0.66	87	0/2
CD62L	-0.58	0.46	87	0/2	-0.58	0.46	87	0/2
CD69	-0.35	0.34	104	0/3	-0.35	0.34	104	0/3
CD94	-1.20	0.40	87	0/2	-1.20	0.40	87	0/2
CXCR6	-1.33	0.71	104	0/3	-1.33	0.71	104	0/3
FAS-L	0.11	0.35	87	0/2	0.11	0.35	87	0/2
FcRg	0.31	0.53	87	0/2	0.31	0.53	87	0/2
HLA-DR	0.05	0.33	87	0/2	0.05	0.33	87	0/2
Ki-67	0.09	0.47	87	0/2	0.09	0.47	87	0/2
NTB-A	-0.44	0.30	87	0/2	-0.44	0.30	87	0/2
PD-1	0.13	0.36	115	1/2	0.14	0.45	87	0/2
Syk	0.11	0.39	87	0/2	0.11	0.39	87	0/2
Tactile	-1.17	1.72	49	0/1	-1.17	1.72	49	0/1
TIGIT	-0.01	0.54	87	0/2	-0.01	0.54	87	0/2
CD57	-1.34	0.63	213	2/3	-1.12	0.39	185	1/3

FR-FCM-ZYZ3 CyTOF Panel

"Cytokine induced changes of NK cells"

Markers	Markers	Markers
A2B4	CD107a	CD14
CD19	CD16	CD161
CD2	CD3	CD4
CD56	CD57	CD69
CD7	CD8	CXCR6
DNAM-1	GM-CSF	GranB
IdU	IFNg	LILRB1
KIR2DL1	KIR2DL2/L3/S2	KIR2DL3
KIR2DL4	KIR2DL5	KIR2DS4
KIR3DL1	KIR3DL1/S1	KIR3DL2
MIP-1b	NKG2A	NKG2C
NKG2D	NKp30	NKp44
NKp46	NTBA	P24
Perforin	TNFa	TRAIL

Cohort	*N*	Conditions
Healthy Donor	8	Unstim, IL-2, IL-15

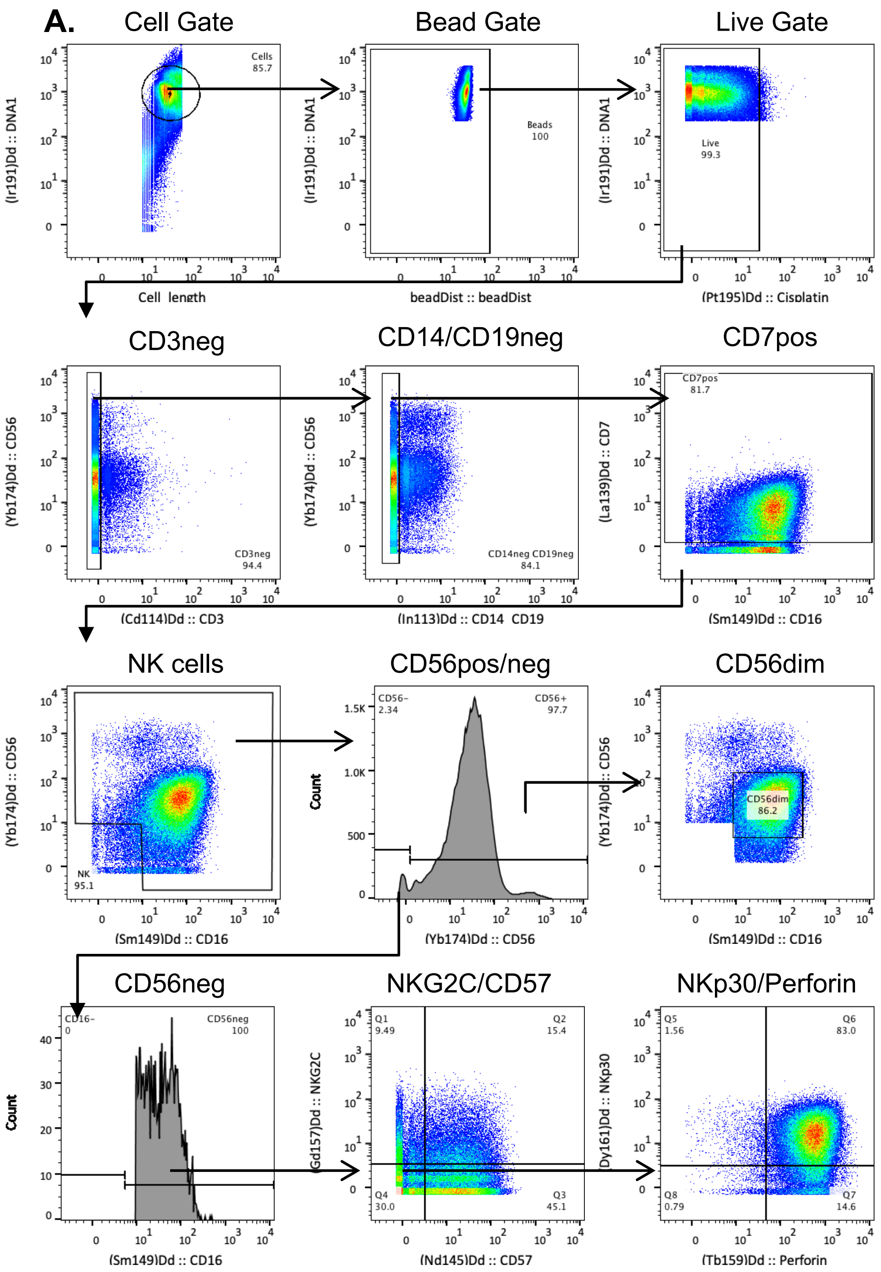

Supplementary Figure 1 – FR-FCM-ZYZ3 CyTOF dataset including IL-2 and IL-15 stimulation conditions

This figure shows the markers labeled, and donors included in the dataset. **A**. shows our gating of these CyTOF files on live $CD3^{-}CD14^{-}CD19^{-}CD7^{+}$ cells, before using CD56 and CD16 to define the CD56dim and CD56neg NK subsets. NKG2C, CD57, NKp30, and Perforin expression were used to define CD56neg cells as either Subset-A ($perforin^{-}NKG2C^{-}NKp30^{-}CD57^{-}$) or Subset-B (all other CD56neg NK cells).

SDY517 CyTOF Panel

"Natural Killer cells in resistance to infection with West Nile virus"

Markers	Markers	Markers
2B4	CD107A	CD14
CD16	CD19	CD3
CD33	CD4	CD56
CD57	CD69	CD7
CD8	CD94	DNAM-1
gdTCR	GM-CSF	HLA-DR
HLA-DR	IdU	IFNg
IL-10	IL-17a	KIR2DL1
KIR2DL3	KIR2DS4	KIR3DL1
LILRB1	MIP-1b	NKG2A
NKG2C	NKG2D	NKp30
NKp44	NKp46	PD-1
Perforin	TNFa	WNV Ag

Cohort	*N*	Conditions
WNV+	3	Unstim, PMA+Io, K562, WNV
Encephalitis	3	
Asymptomatic	8	

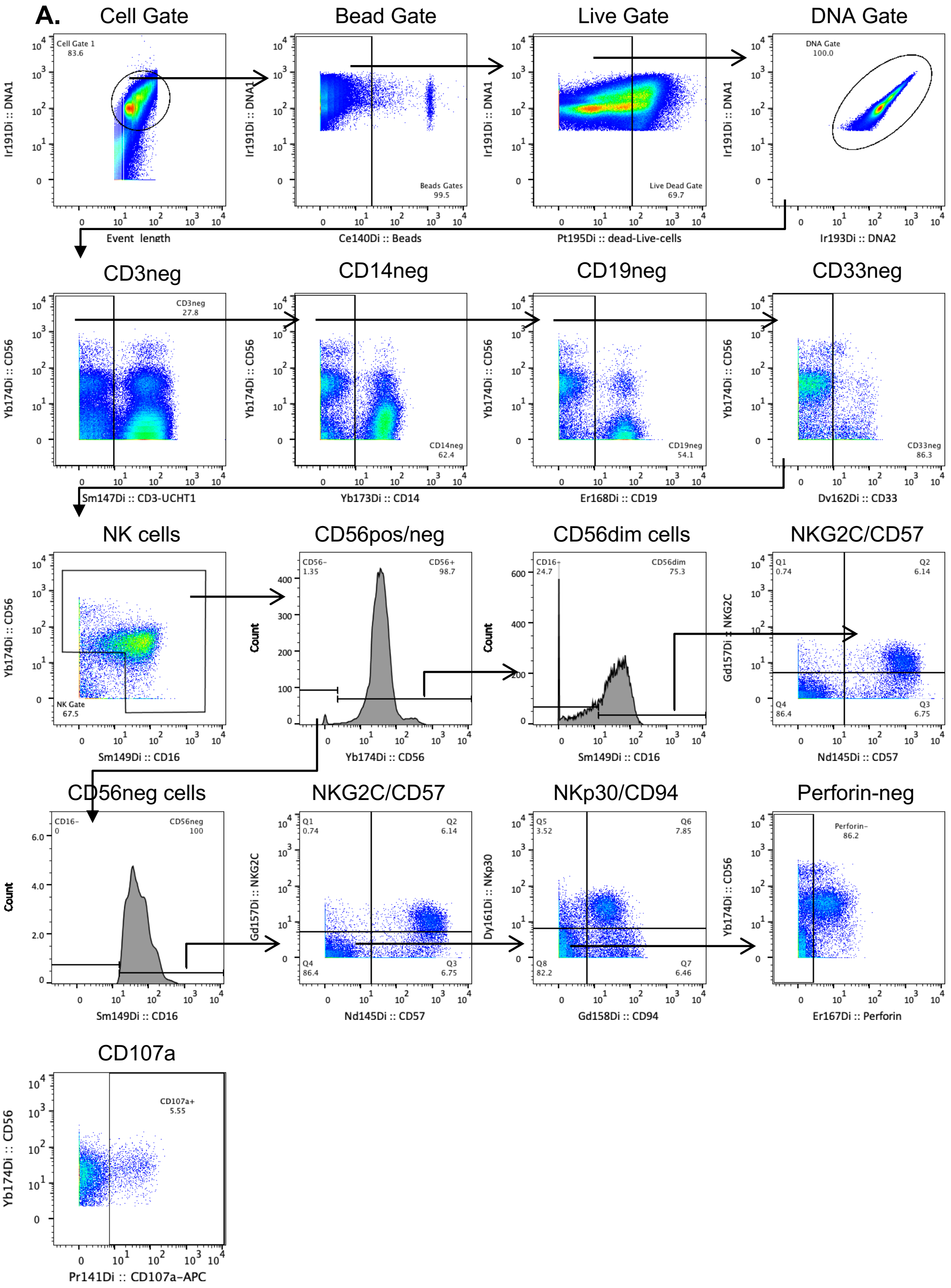

Supplementary Figure 2 – SDY517 CyTOF dataset including K562 stimulation

This figure shows the markers labeled, and donors included in the dataset. **A**. shows our gating of these CyTOF files on live $CD3^{-}CD14^{-}CD19^{-}CD33^{-}$ cells, before using CD56 and CD16 to define the CD56dim and CD56neg NK subsets. NKG2C, CD57, NKp30, CD94, and Perforin expression were used to define CD56neg cells as either Subset-A ($perforin^{-}CD94^{-}NKG2C^{-}NKp30^{-}CD57^{-}$) or Subset-B (all other CD56neg NK cells). CD107a gating is also shown.

SDY1535 CyTOF Panel

"TIGIT is upregulated by HIV-1 infection and marks a highly functional adaptive and mature subset of natural killer cells"

Markers	Markers	Markers
2B4	CD14	CD33
CD16	CD19	CD2
CD3	CD38	CD4
CD56	CD57	CD62L
CD69	CD8	CD94
CD96	CXCR6	DNAM-1
FAS-L	FcRg	HLA-DR
Ki-67	KIR2DL1	KIR2DL3
KIR3DL5	KIR2DS2	KIR2DS4
KIR3DL1	LILRB1	NKG2A
NKG2C	NKG2D	NKp30
NKp44	NKp46	NTB-A
PD-1	Perforin	Siglec-7
Syk	TIGIT	

Cohort	*N*	Conditions
HIV-ART	20	Unstim
HIV-NoART	20	
HIV-Neg	10	

A.

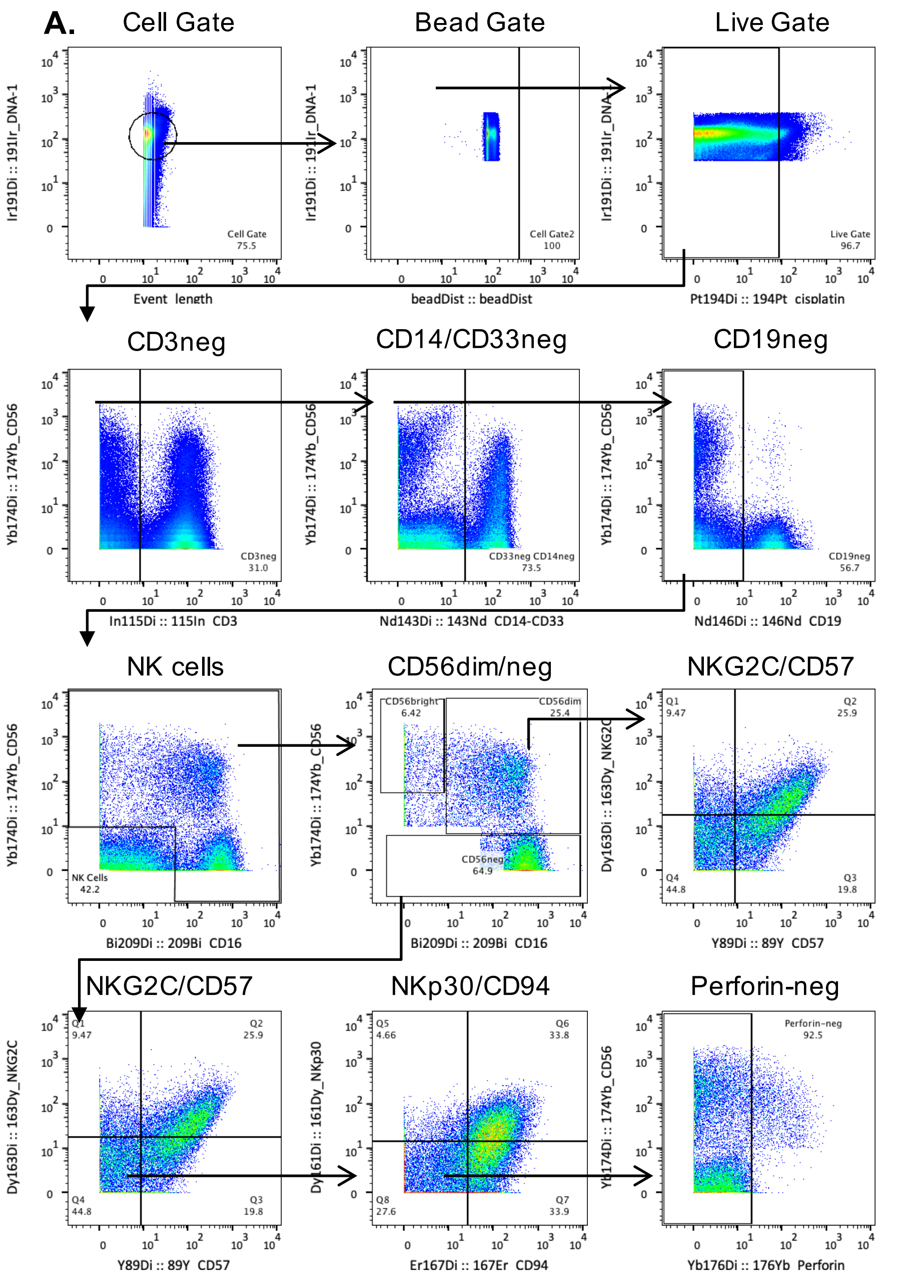

Supplementary Figure 3 – SDY1535 CyTOF dataset including HIV-1 positive and HIV-1 negative samples

This figure shows the markers labeled, and donors included in the dataset. **A**. shows our gating of these CyTOF files on live $CD3^{-}CD14^{-}CD19^{-}CD33^{-}$ cells, before using CD56 and CD16 to define the CD56dim and CD56neg NK subsets. NKG2C, CD57, NKp30, CD94, and Perforin expression were used to define CD56neg cells as either Subset-A ($perforin^{-}CD94^{-}NKG2C^{-}NKp30^{-}CD57^{-}$) or Subset-B (all other CD56neg NK cells).

SDY1603 CyTOF Panel

“Investigating the natural killer cell response to acute dengue infection.”

Markers	Markers	Markers
2B4	CD16	CD19
CD2	CD3	CD33
CD14	CD38	CD4
CD45	CD56	CD57
CD62L	CD69	CD8
CD94	CXCR6	DNAM-1
FAS-L	FcRg	HLA-DR
Ki-67	KIR2DL1	KIR2DL3
KIR2DL5	KIR2DS2	KIR2DS4
KIR3DL1	LILRB1	NKG2A
NKG2C	NKG2D	NKp30
NKp44	NKp46	NTB-A
PD-1	Perforin	Siglec-7
Syk	Tactile	TIGIT

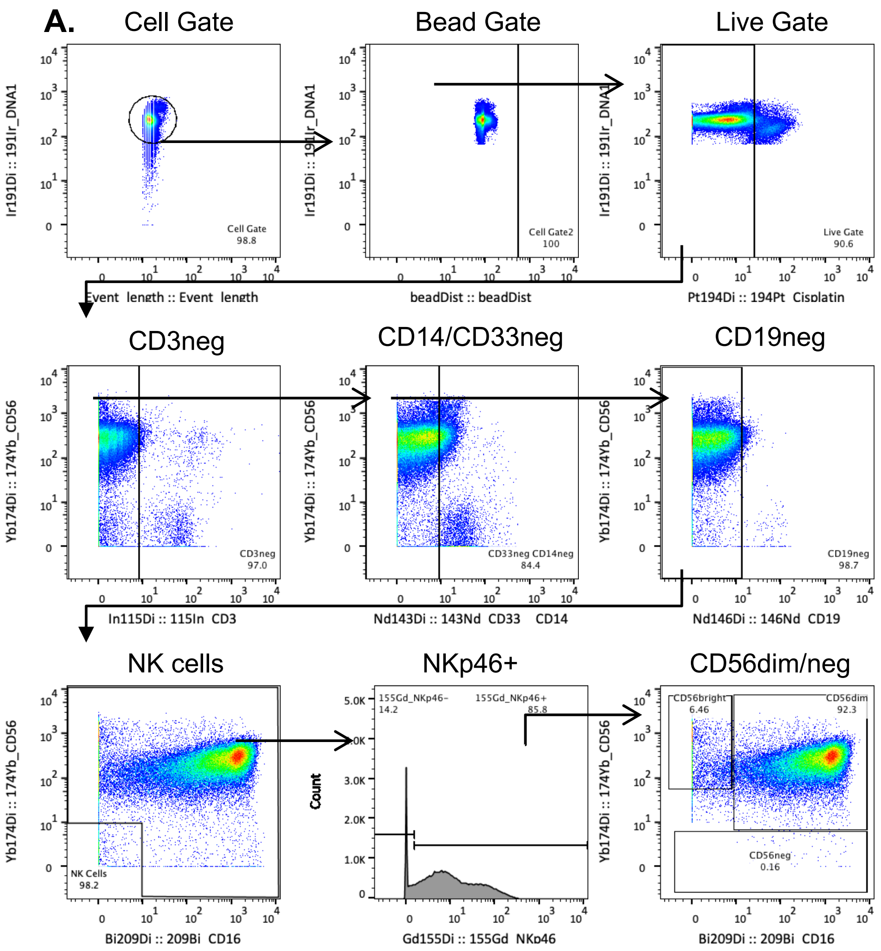

Cohort	*N*	Conditions
Adult acute dengue	7	Unstim
Adult healthy	31	
Paediatric acute dengue	8	
Paediatric healthy	21	

Supplementary Figure 4 – SDY1603 CyTOF dataset including dengue positive negative adult and pediatric samples

This figure shows the markers labeled, and donors included in the dataset. **A**. shows our gating of these CyTOF files on live $CD3^-CD14^-CD19^-CD33^-NKp46^+$ cells, before using CD56 and CD16 to define the CD56dim and CD56neg NK subsets.

FR-FCM-ZYZX CyTOF Panel

"Data from Simoni et al Immunity 2017"

Markers	Markers	Markers
CD45	CD14	CD27
CD4	HLA-DR	NKG2D
NKp80	CD16	CD69
CD8a	CD34	CCR4
CCR6	KLRG1	CD5
CD122	CD103	CXCR6
Tbet	FceRIa	CD19
CD123	Granzyme A	CD56
CD161	NKp44	NKp30
KI-67	CD127	Granzyme B
ICOS	NKp46	c-Kit
CCR7	CD25	2B4
CRTH2	CD94	IL-23R
CD160	Perforin	EOMES

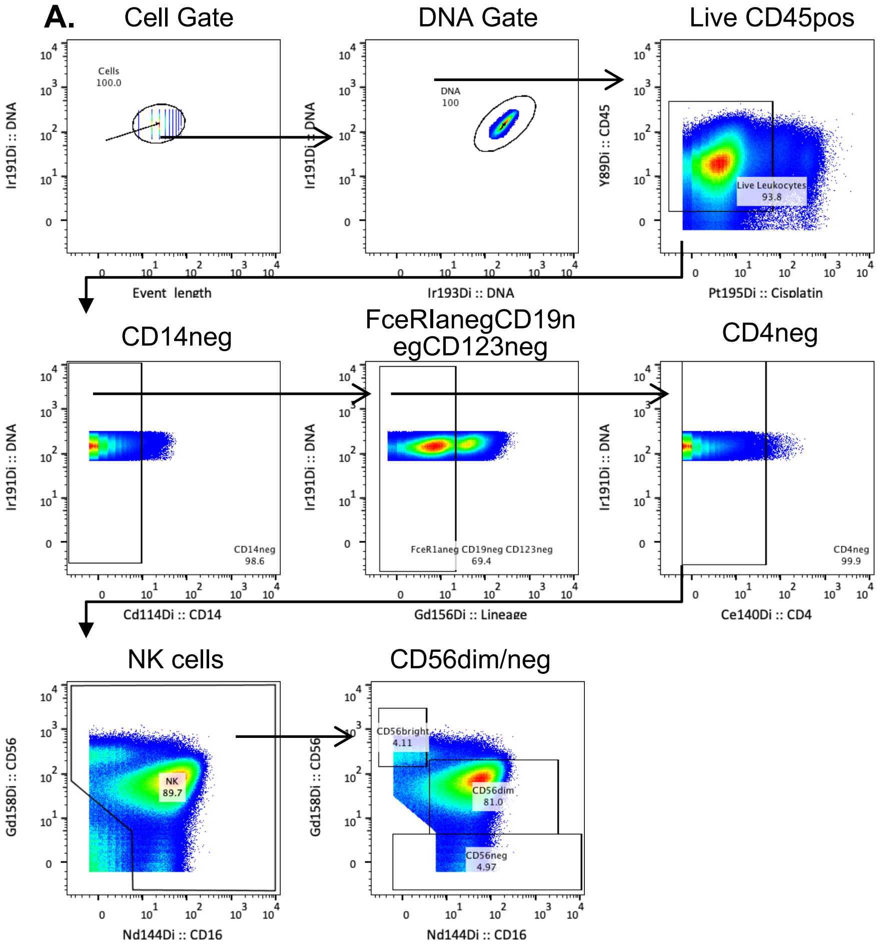

Cohort	*N*	Conditions
Bonemarrow	2	Unstim
Colon	3	
Colorectal tumour	4	
Cord blood	4	
Lung	3	
Lung Tumour	3	
Omentum	2	
Blood	19	
Skin	2	
Spleen	3	
Tonsil	5	

Supplementary Figure 5 – FR-FCM-ZYZX CyTOF dataset including cells from multiple tissue samples

This figure shows the markers labeled, and tissues included in the dataset. **A**. shows our gating of these CyTOF files on live $CD45^{+}CD14^{-}CD19^{-}CD123^{-}CD4^{-}$ cells, before using CD56 and CD16 to define the CD56dim and CD56neg NK subsets.

FR-FCM-ZY9N CyTOF Panel

"CyTOF analysis of paired blood, tumor and non-involved lung from NSCLC patients"

Markers	Markers	Markers
CD45	FceRIa	HLA-ABC
CD19	CD117	CD141
CCR2	CD64	GATA6
CD16	CD123	CD66b
CD1c	CD163	CD86
CD33	CD14	CD11c
CD115	CCR3	CD116
CD24	CD206	CD3
CD68	CD11b	HLA-DR
PD-L1	CD56	

Cohort	*N*	Conditions
Blood	20	Ustim NSCLC patients
Lung	20	
Lung tumour	20	

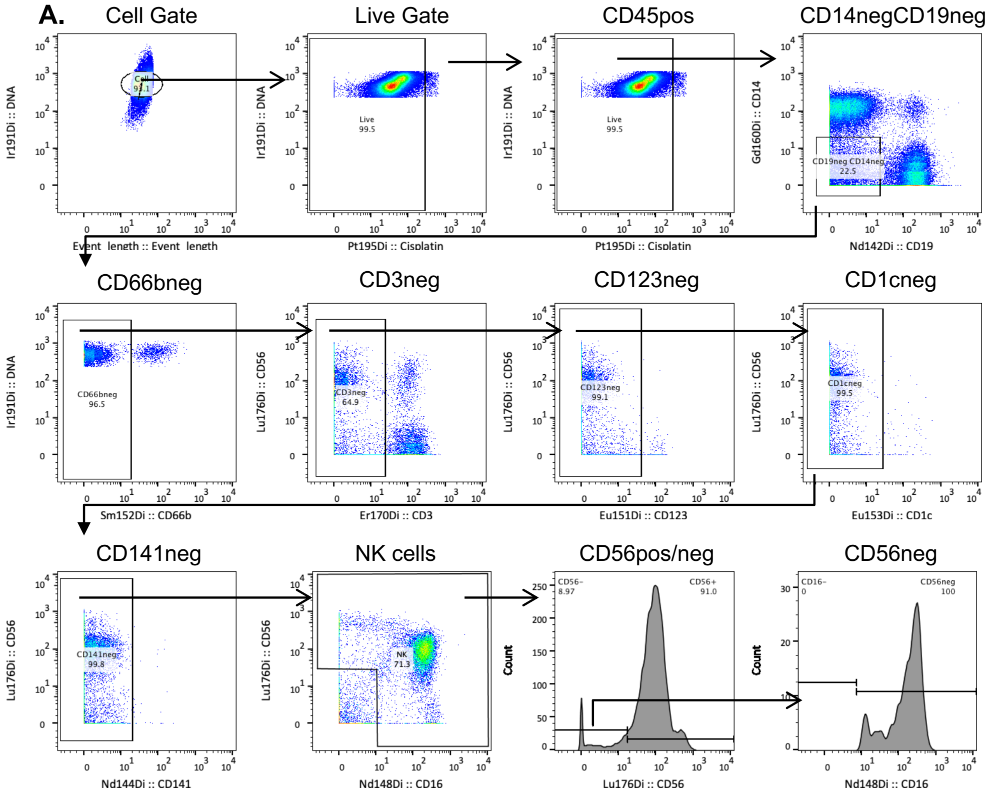

Supplementary Figure 6 – FR-FCM-ZYZN CyTOF dataset including cells from lung and paired tumor samples

This figure shows the markers labeled, and donors included in the dataset. **A**. shows our gating of these CyTOF files on live CD45^{+}CD1c^{-}CD3^{-}CD14^{-}CD19^{-}CD66b^{-}CD123^{-}CD141^{-} cells, before using CD56 and CD16 to define the CD56dim and CD56neg NK subsets.

FR-FCM-Z2XC CyTOF Panel

"Demonstration of a whole blood CyTOF staining workflow to characterize COVID-19 patients"

Markers	Markers	Markers
CD45	Granzyme B	IgA
CD11b	IgM	CD86
IgG	CCR6	ICOS
CD123	CD19	CD4
CD8a	CD11c	CD16
CD45RO	CD45RA	CD161
CCR4	CD25	CD27
CD57	CXCR3	CXCR5
SARS-S	CD28	CD38
CD169	CD56	TCRgd
SARS-NP	CD294	CCR7
CD14	CD71	CD3
CD20	CD66b	HLA-DR
IgD	PD-1	CD127
CD61		

Cohort	*N*	Conditions
Healthy	7	Unstim
Covid patient	14	

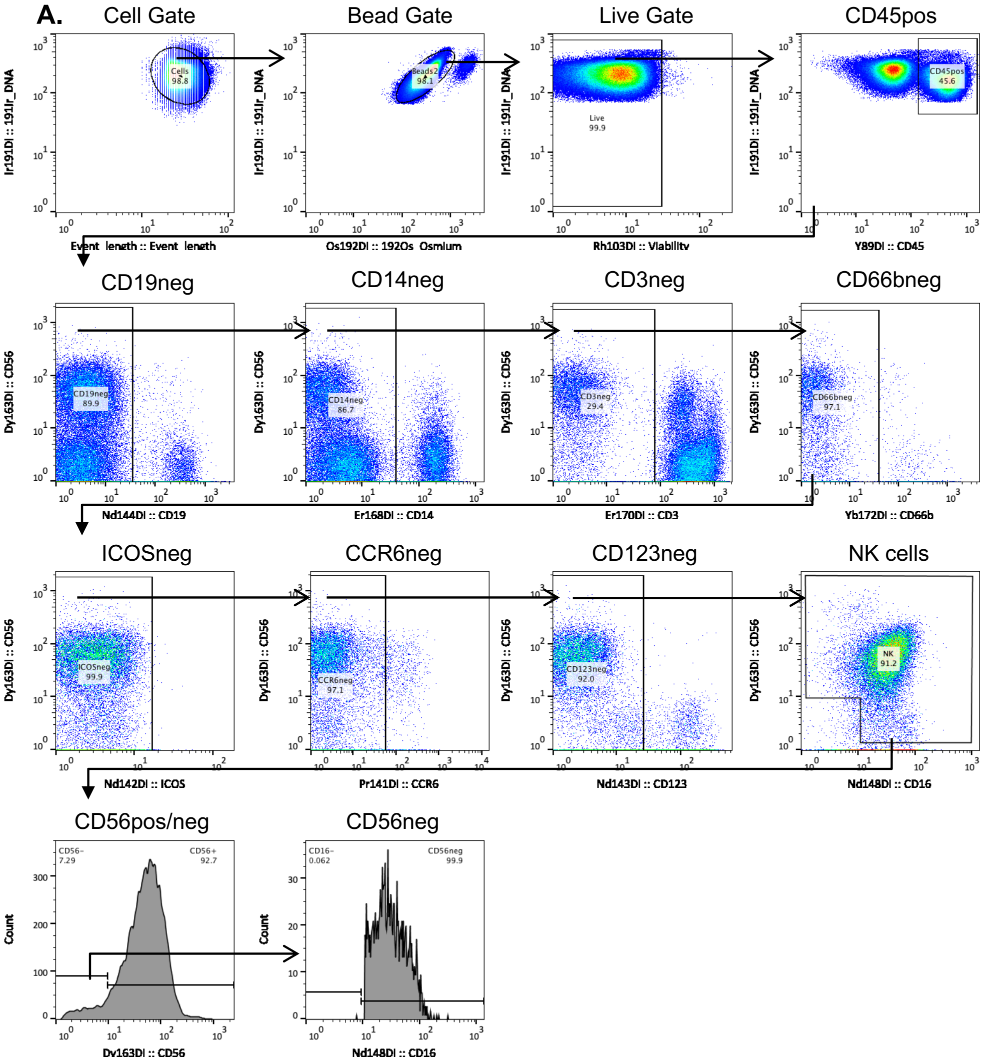

Supplementary Figure 7 – FR-FCM-Z2XC CyTOF dataset including cells from covid-19 patient samples

This figure shows the markers labeled, and donors included in the dataset. **A**. shows our gating of these CyTOF files on live $CD45^{+}CD3^{-}CD14^{-}CD19^{-}CD66b^{-}ICOS^{-}CCR6^{-}CD123^{-}$ cells, before using CD56 and CD16 to define the CD56dim and CD56neg NK subsets.

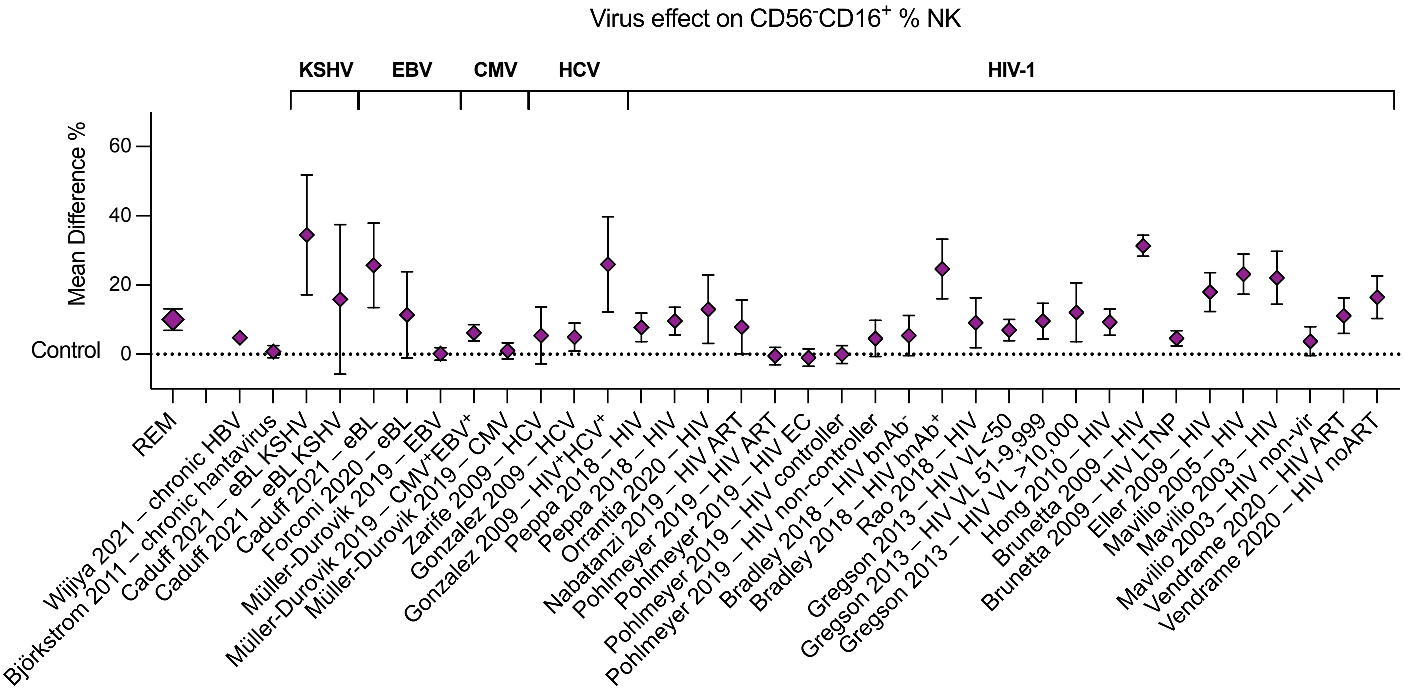

Supplementary Figure 8 – Meta-analysis of CD56neg subset frequency in individuals with chronic virus infections

This graph shows mean difference of CD56neg % NK cells in peripheral blood in individuals with chronic virus infections from study controls (REM mean difference 10.05%, CI ± 3.11, $I^2 = 94\%$; study n=18, dataset n=1, combined chronic virus samples n=818, combined study control samples = 702).

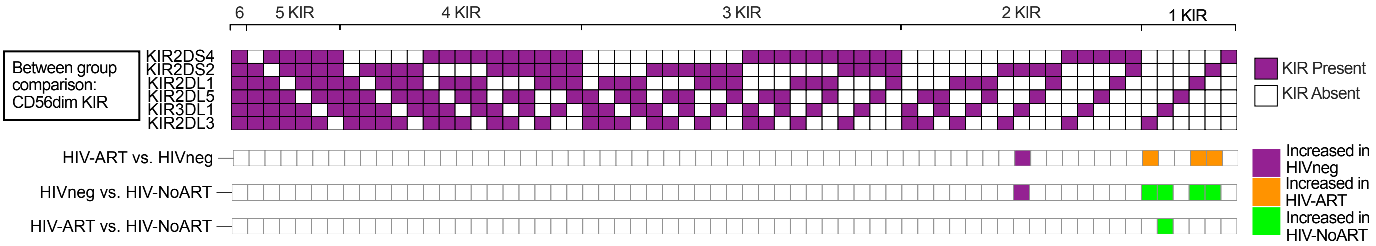

Supplementary Figure 9 – HIV-1 status affects the KIR combinations of CD56dim NK cells

This heatmap explores the impact of HIV-1 and ART status on KIR combination frequency within the CD56neg subset through between-group comparisons (SDY1535). KIR combinations that were significantly increased in the CD56neg cells of HIV negative individuals were shown as purple squares, those increased in those of HIV-ART group were denoted as orange, and those increased in HIV-NoART were depicted as green. Two-way ANOVA results with multiple comparisons corrected using the Sídák method are shown.

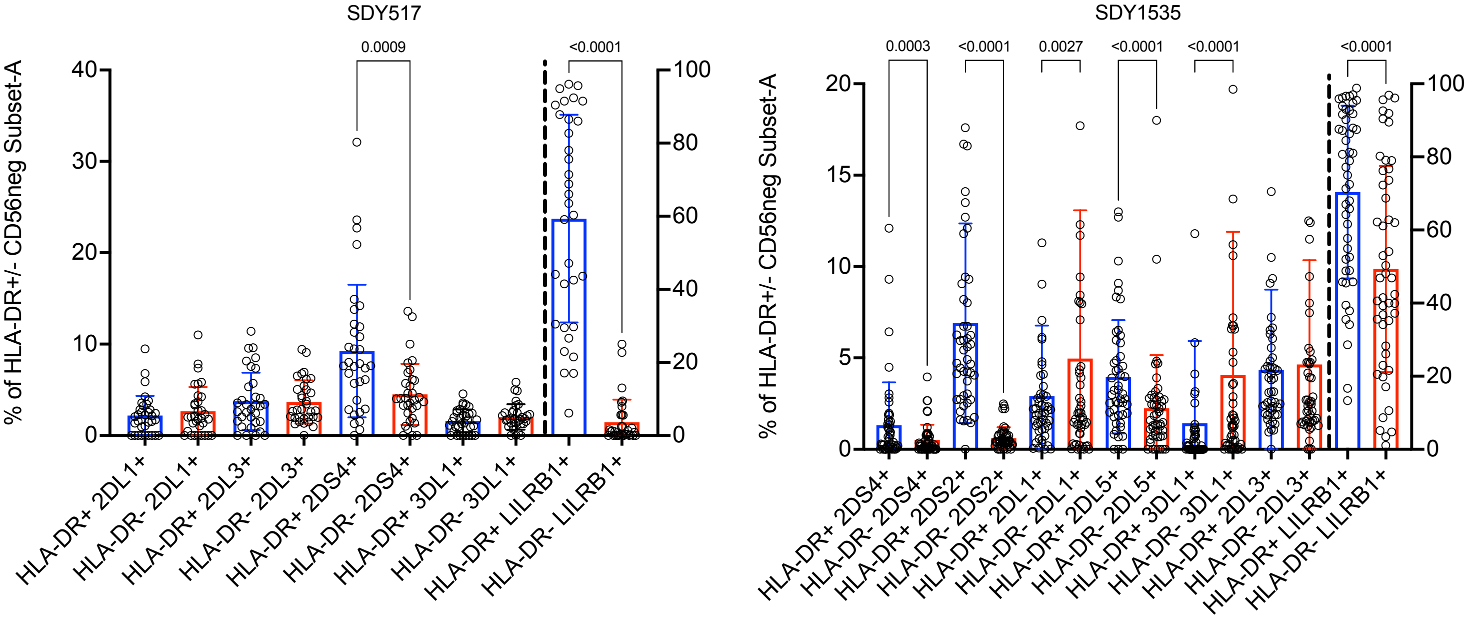

Supplementary Figure 10 – KIR expression comparison between HLA-DR^{+} and HLA-DR^{-} Subset-A CD56neg NK cells

These graphs show the frequency of KIR, and LILRB1, expressing HLA-DR positive and negative populations that make up CD56neg Subset-A NK cells from the SDY517 and SDY1535 CyTOF datasets. If the HLA-DR expression on Subset-A CD56neg cells was due to to contaminating dendritic cells we would not expect the HLA-DR positive cells to express KIR receptors, however we see here that Subset-A CD56neg cells contain several populations that co-express HLA-DR and KIRs. Analysis used two-way ANOVA with multiple comparisons corrected using the Sídák method. Mean and SD are shown.

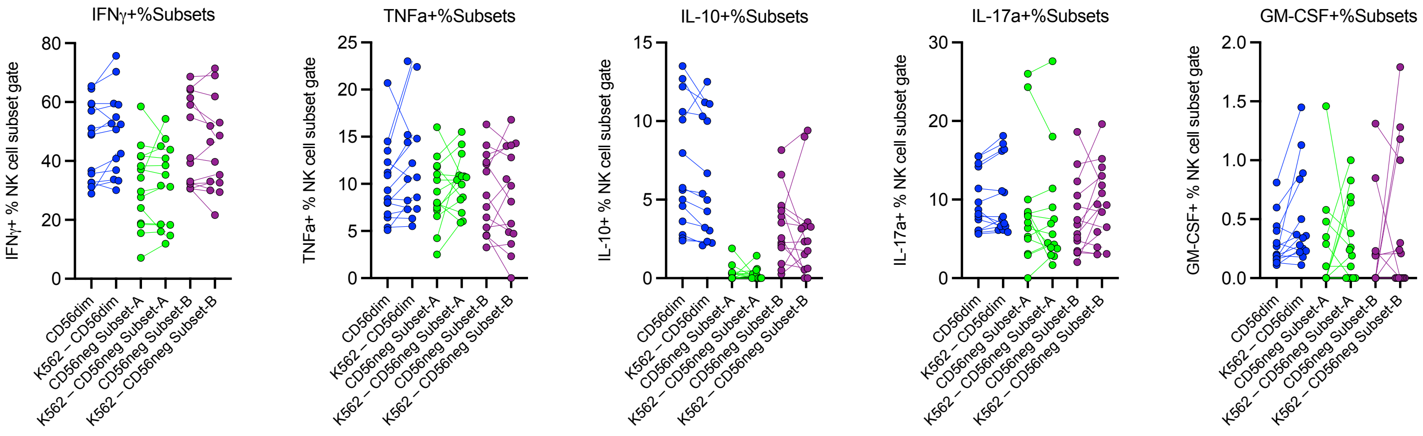

Supplementary Figure 11 – Comparison of cytokine expression related to CD56dim, and CD56neg Subset-A and -B NK cells

These graphs show the mean differences in IFNγ^{+}, IL-10^{+}, GM-CSF^{+}, IL-17a^{+}, and TNFa^{+} CD56dim, CD56neg Subset-A and -B cell frequencies (SDY517). Friedman tests corrected using the FDR method were used to assess within subset expression change.

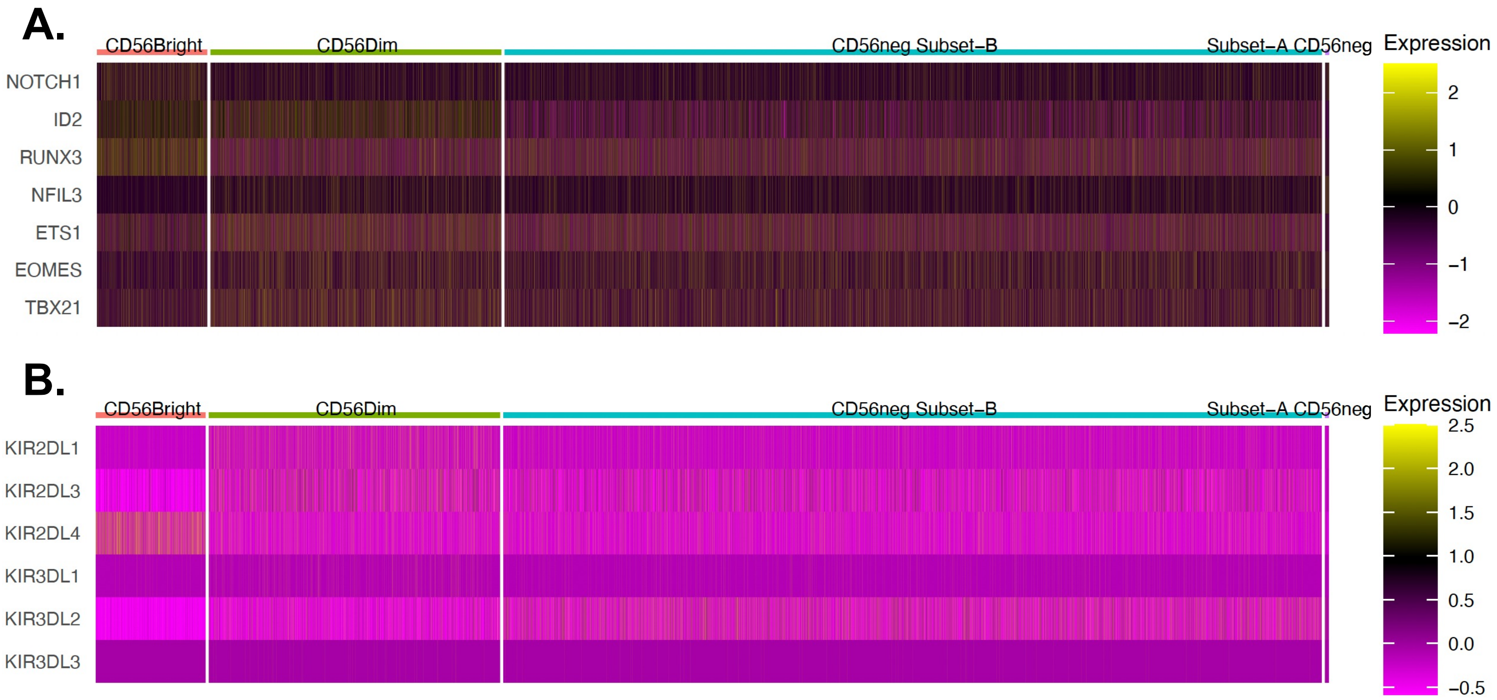

Supplementary Figure 12 – Comparison of gene expression related to NK development and DC subsets Plot **A.** shows the expression level of maturation associated genes for the CD56bright, CD56dim, CD56neg Subset-A and -B NK cell subsets. **B.** shows the expression level of KIR genes for these same NK subsets.
