# Supplementary material for: CD56-negative NK cells: Frequency in peripheral blood, expansion during HIV-1 infection, functional capacity, and KIR expression
Source: Front Immunol. 2022 Sep 23;13:992723. doi: 10.3389/fimmu.2022.992723 (PMC9539804; doi:10.3389/fimmu.2022.992723)
Supplement: Supplementary file 2 [file Presentation_1.pptx]

## Slide 1
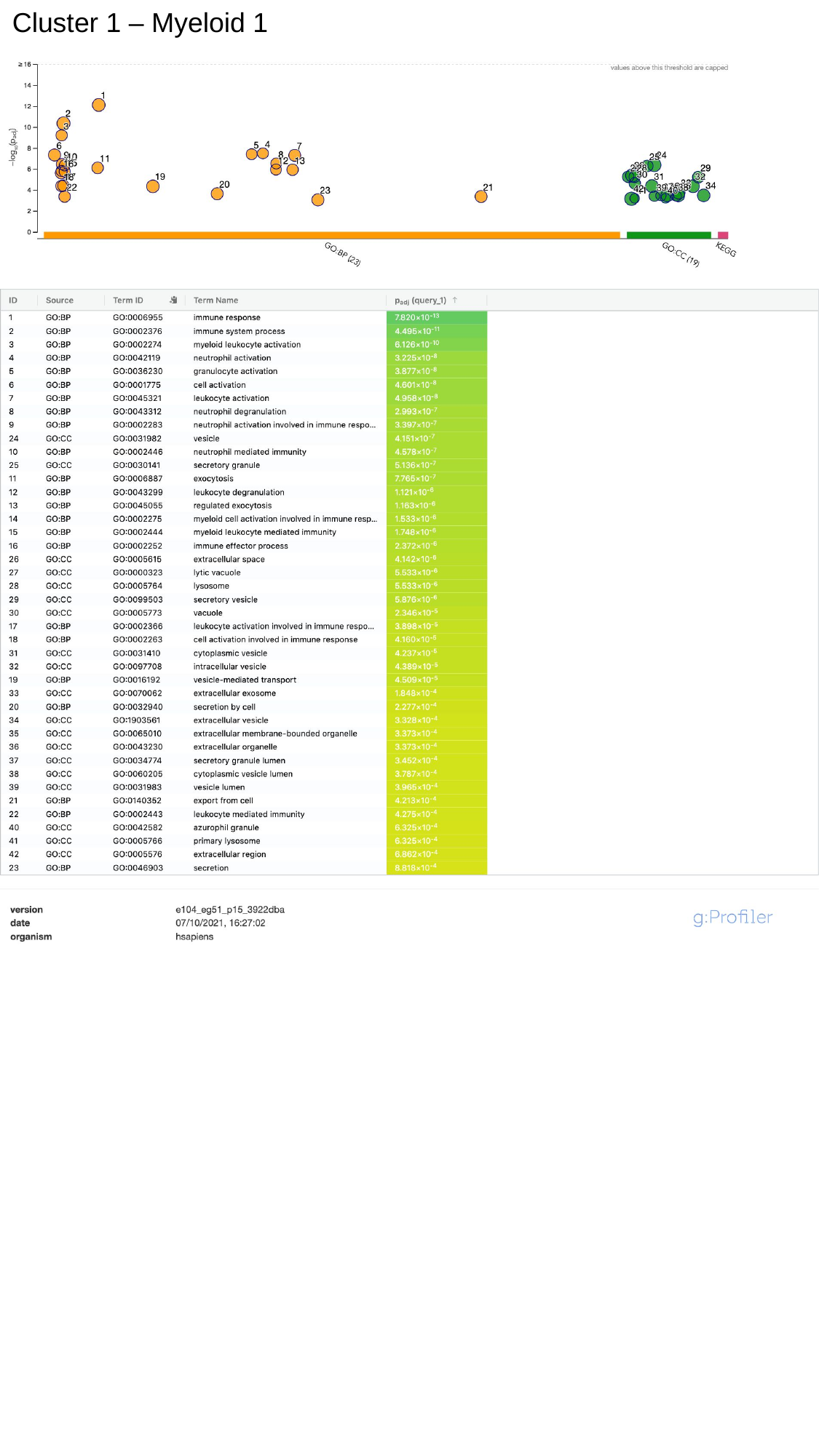

Cluster 1 – Myeloid 1

## Slide 2
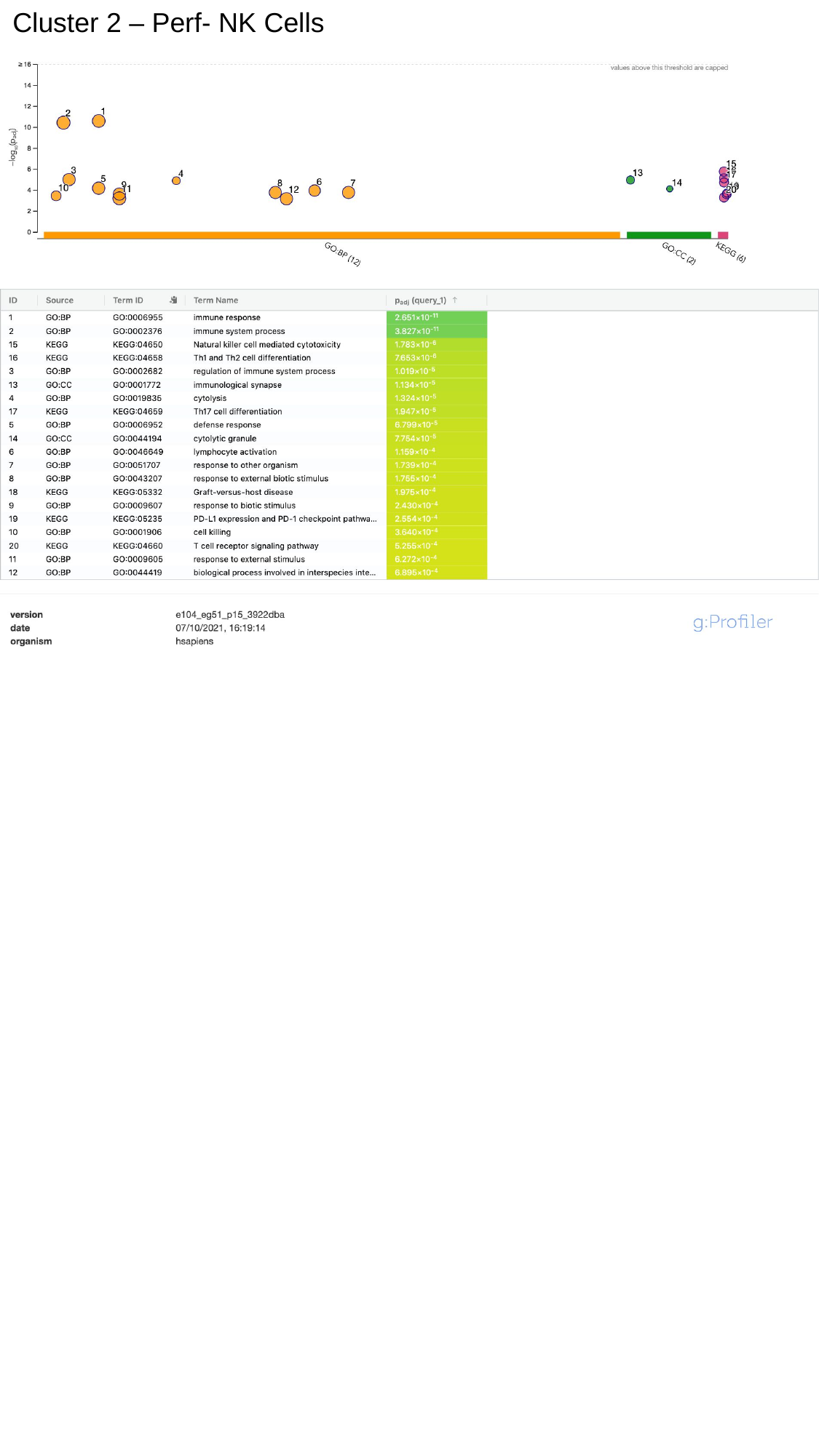

Cluster 2 – Perf- NK Cells

## Slide 3
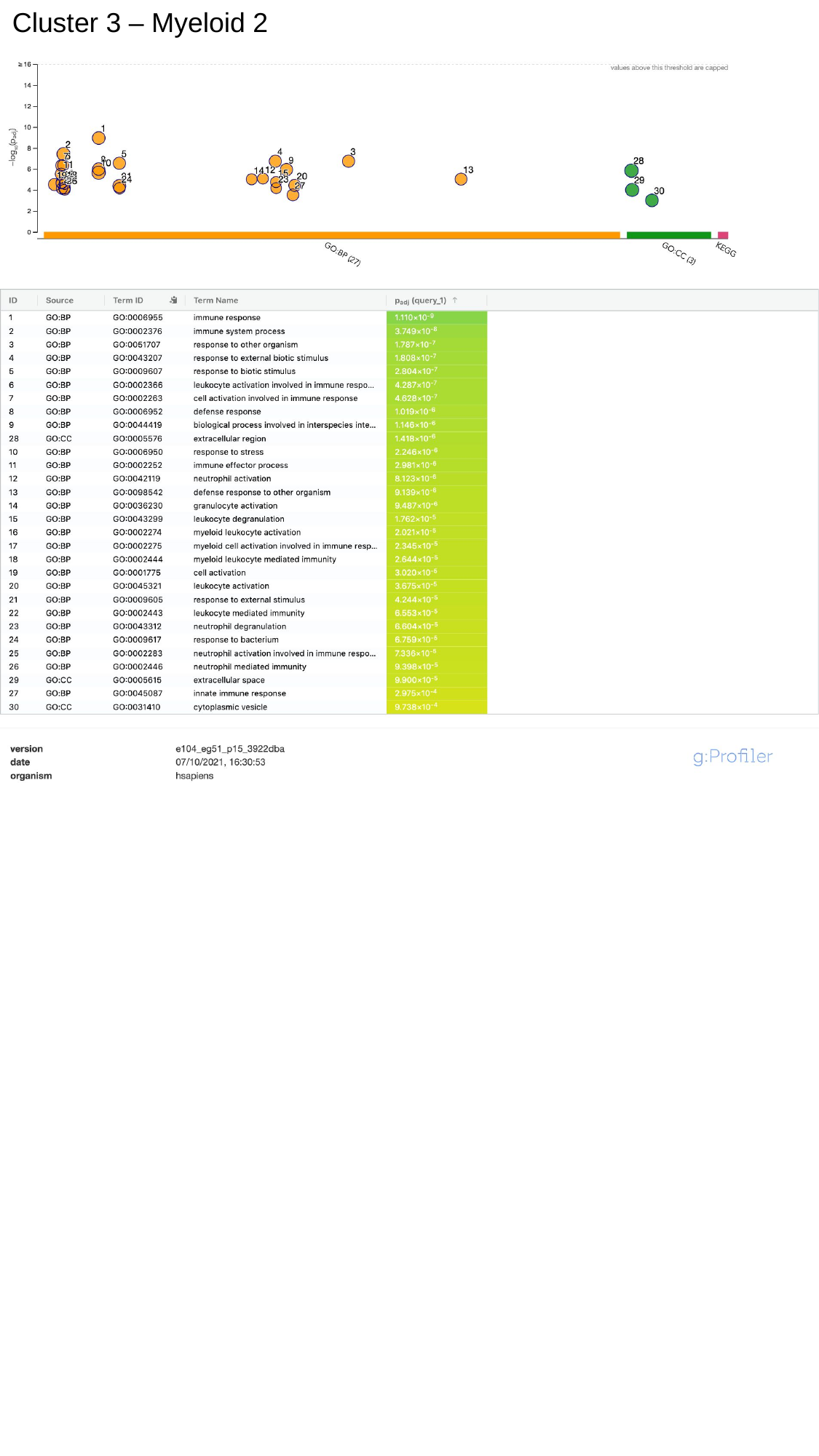

Cluster 3 – Myeloid 2

## Slide 4
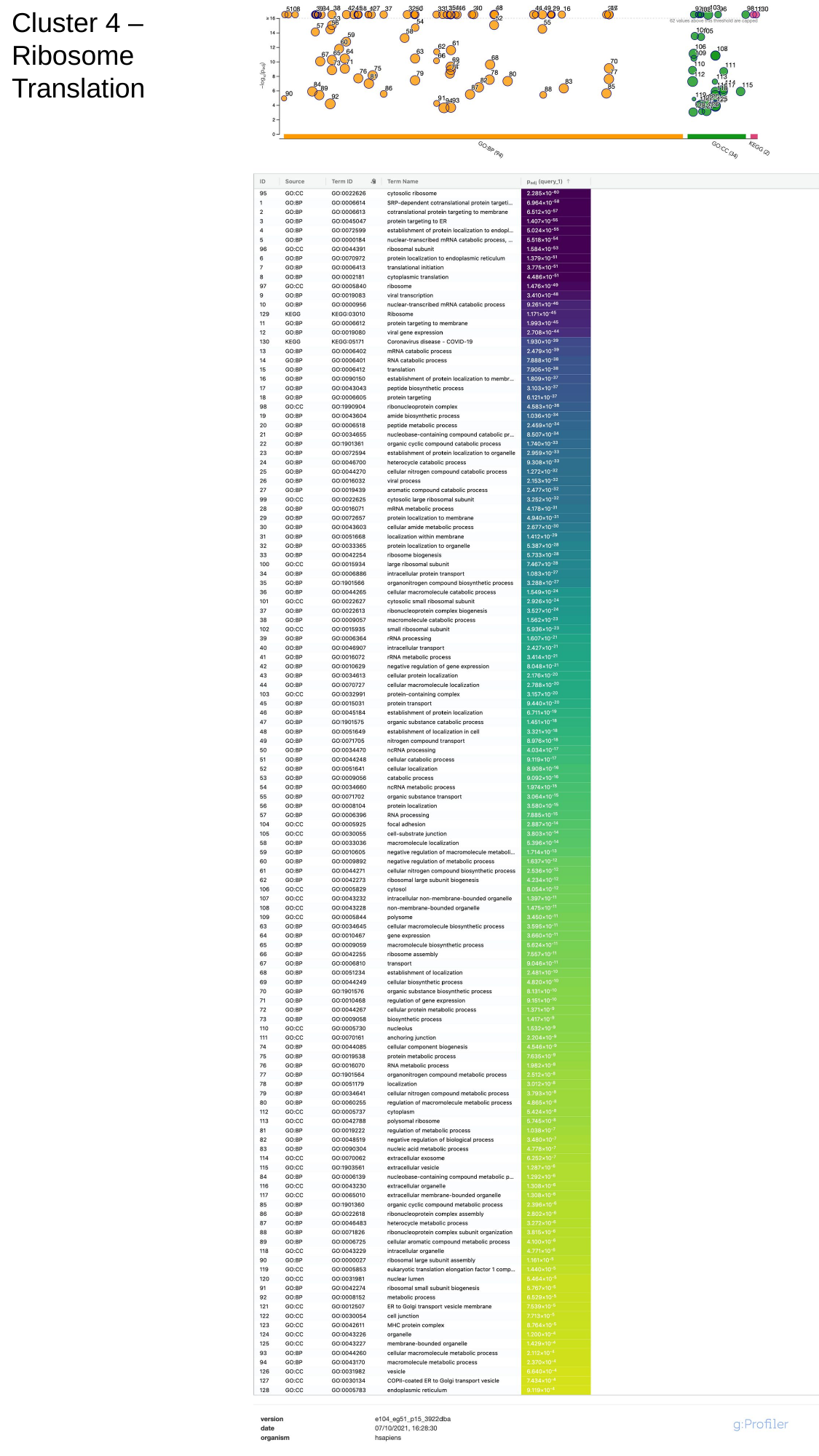

Cluster 4 –
Ribosome
Translation

## Slide 5
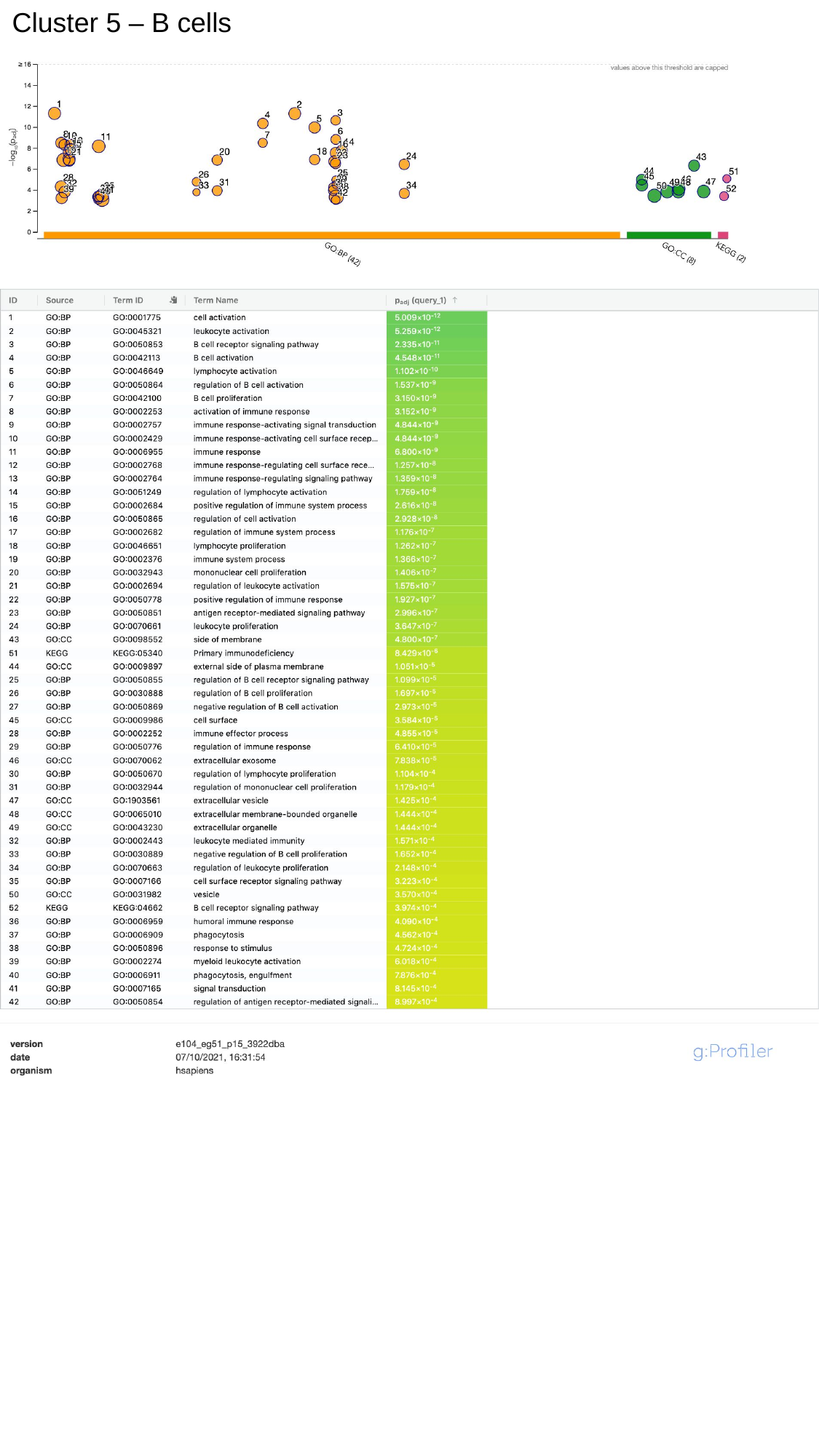

Cluster 5 – B cells

## Slide 6
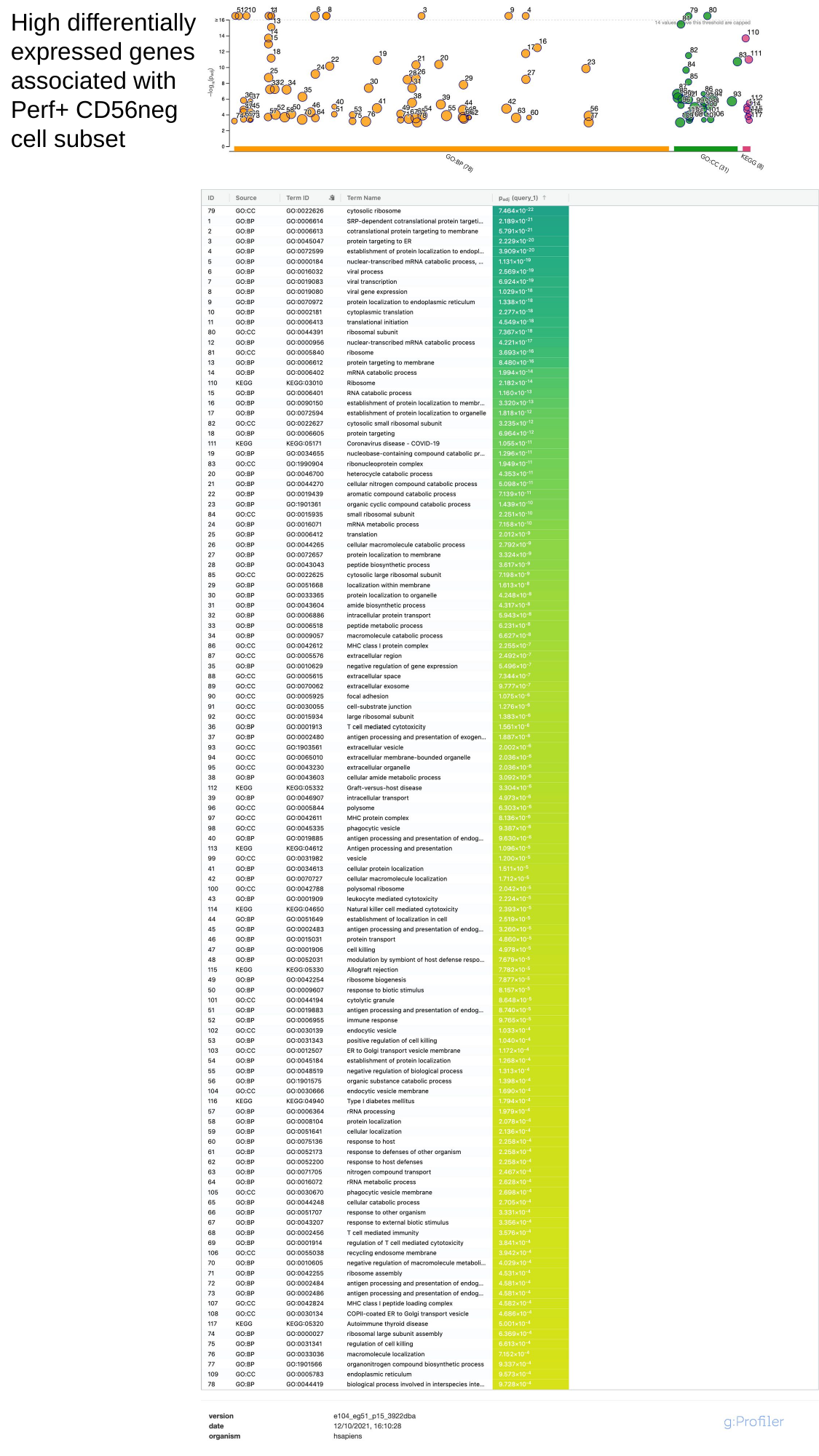

High differentially expressed genes associated with Perf+ CD56neg cell subset

## Slide 7
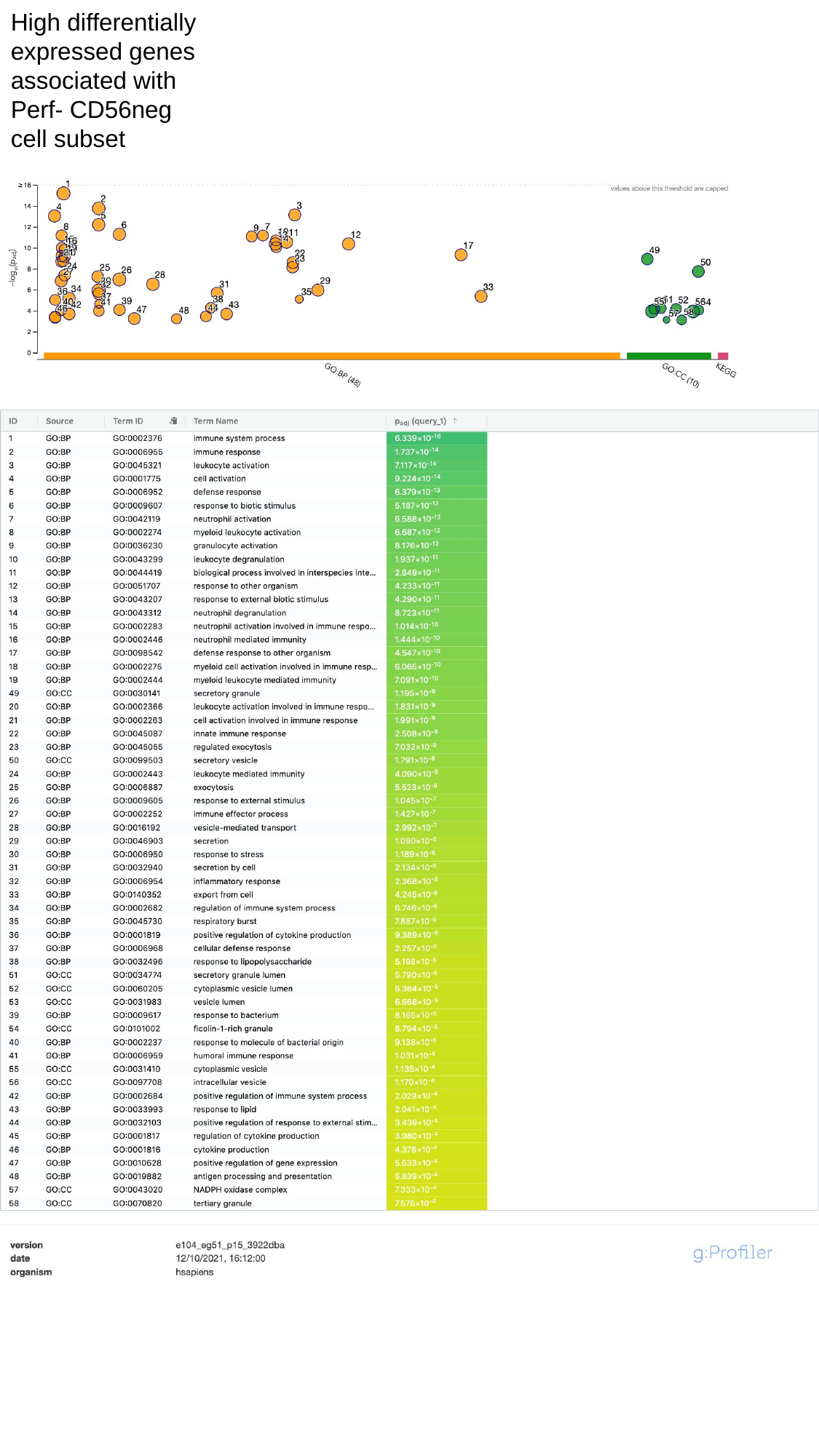

High differentially expressed genes associated with Perf- CD56neg cell subset
